# Safety and Immunogenicity of DNA and MVA HIV-1 Subtype C Vaccine Prime-Boost Regimens: A Phase I Randomised Trial in HIV-Uninfected Indian Volunteers

**DOI:** 10.1371/journal.pone.0055831

**Published:** 2013-02-13

**Authors:** Sanjay Mehendale, Madhuri Thakar, Seema Sahay, Makesh Kumar, Ashwini Shete, Pattabiraman Sathyamurthi, Amita Verma, Swarali Kurle, Aparna Shrotri, Jill Gilmour, Rajat Goyal, Len Dally, Eddy Sayeed, Devika Zachariah, James Ackland, Sonali Kochhar, Josephine H. Cox, Jean-Louis Excler, Vasanthapuram Kumaraswami, Ramesh Paranjape, Vadakkuppatu Devasenapathi Ramanathan

**Affiliations:** 1 National Institute of Epidemiology, Chennai, India; 2 National AIDS Research Institute, Pune, India; 3 National Institute for Research on Tuberculosis, Chennai, India; 4 International AIDS Vaccine Initiative, Delhi, India; 5 IAVI Human Immunology Laboratories, Imperial College, London, United Kingdom; 6 The EMMES Corporation, Rockville, Maryland, United States of America; 7 InternationalAIDS Vaccine Initiative, New York, New York, United States of America; 8 Global BioSolutions, Melbourne, Victoria, Australia; 9 OneWorldHealth, New Delhi, India; Naval Medical Research Center, United States of America

## Abstract

**Study Design:**

A randomized, double-blind, placebo controlled phase I trial.

**Methods:**

The trial was conducted in 32 HIV-uninfected healthy volunteers to assess the safety and immunogenicity of prime-boost vaccination regimens with either 2 doses of ADVAX, a DNA vaccine containing Chinese HIV-1 subtype C *env gp160, gag, pol* and *nef/tat* genes, as a prime and 2 doses of TBC-M4, a recombinant MVA encoding Indian HIV-1 subtype C *env gp160, gag, RT, rev, tat*, and *nef* genes, as a boost in Group A or 3 doses of TBC-M4 alone in Group B participants. Out of 16 participants in each group, 12 received vaccine candidates and 4 received placebos.

**Results:**

Both vaccine regimens were found to be generally safe and well tolerated. The breadth of anti-HIV binding antibodies and the titres of anti-HIV neutralizing antibodies were significantly higher (p<0.05) in Group B volunteers at 14 days post last vaccination. Neutralizing antibodies were detected mainly against Tier-1 subtype B and C viruses. HIV-specific IFN-γ ELISPOT responses were directed mostly to Env and Gag proteins. Although the IFN-γ ELISPOT responses were infrequent after ADVAX vaccinations, the response rate was significantly higher in group A after 1^st^ and 2^nd^ MVA doses as compared to the responses in group B volunteers. However, the priming effect was short lasting leading to no difference in the frequency, breadth and magnitude of IFN-γELISPOT responses between the groups at 3, 6 and 9 months post-last vaccination.

**Conclusions:**

Although DNA priming resulted in enhancement of immune responses after 1^st^ MVA boosting, the overall DNA prime MVA boost was not found to be immunologically superior to homologous MVA boosting.

**Trial Registration:**

Clinical Trial Registry CTRI/2009/091/000051

## Introduction

A safe and efficacious HIV vaccine is urgently needed to curtail the HIV pandemic. India is currently facing a burden of 2.39 million people living with HIV/AIDS, although the estimated HIV prevalence in the adult population is only 0.31% [1]. For effective control of HIV/AIDS in India, an HIV vaccine may prove to be a useful addition to other available prevention options. Two phase I clinical HIV prophylactic vaccine trials have been conducted previously in India to evaluate Adenovirus-Associated Virus (AAV) and Modified Vaccinia Ankara (MVA) based HIV vaccines. Although the AAV-based vaccine showed poor immunogenicity, the MVA HIV-1 subtype C vaccine induced a modest level of dose-dependent immune responses [2], [3], [4].

Since vaccine strategies based on inducing neutralizing antibodies failed in large scale phase III trials [5], [6] the direction of HIV prophylactic vaccine research shifted to evaluating vaccine candidates having the ability to induce cell-mediated immune responses. However, a higher magnitude and limited breadth of T-cell responses, as detected by Interferon-gamma (IFN-γ) ELISPOT assays, did not correlate with protection in monkey models [7].

A phase III clinical trial (RV144) is the only large scale HIV vaccine trial that demonstrated a modest reduction in the infection rates among the vaccinees. The trial used a heterologous prime-boost regimen consisting of a recombinant canarypox vector prime followed by recombinant Env gp120 protein boost [8]. The major advantage of heterologous boosting with vector based vaccines is the obviation of vector-induced immune responses after repeated doses of the same construct affecting generation of immune responses against target antigens [9], [10]. Heterologous boosting also provides potential for different vectors to work synergistically by stimulating complementary arms of the immune response [9]. Among different combinations of heterologous vaccinations, plasmid DNA with one or more viral vectors has been studied most extensively in various preclinical and clinical trials [11], [12], [13], [14], [15], [16]. Although DNA constructs themselves have been shown to induce weak immune responses, subsequent heterologous boosting with viral vectors has been shown to induce potent antibody and cell-mediated immune responses [13], [14], [15]. DNA vaccinations have also been shown to confer partial protection in terms of reduction in viremia in vaccinated macaques challenged with Simian Immuno-deficiency Virus [SIV] or Simian/Human Immuno-deficiency Virus [SHIV], despite their low immunogenicity [17], [18]. Vaccine strategies with DNA priming followed by boosting with a recombinant MVA vector encoding the same immunogen have been attempted against several diseases, including HIV [16], [19], [20], [21], [22] malaria [23] tuberculosis [24] and cancer [25].

The phase I HIV-1 subtype C prophylactic vaccine trial described in this report was conducted in Pune and Chennai in India. It was designed to assess the safety and immunogenicity of a heterologous prime-boost immunization regimen using DNA prime and MVA boost versus the homologous prime and boost with MVA alone.

## Materials and Methods

The protocol for this trial and supporting CONSORT checklist are available as supporting information; see Checklist S1 and Protocol S1.

### Ethics Statement

The study protocol was approved by the Central Drug and Standards Control Organization (formerly Drugs Controller General of India) as well as by the Institutional Ethics Committees and Scientific Advisory Committees of the National AIDS Research Institute (NARI) and of the National Institute for Research in Tuberculosis (NIRT, formerly Tuberculosis Research Centre-TRC). The study was conducted in accordance with International Conference on Harmonization - Good Clinical Practice (ICH-GCP) and Good Clinical Laboratory Practice (GCLP). All participants provided written informed consent.

### Candidate Vaccines

ADVAX (Lot # 04030248, Vical, Inc., San Diego, CA), is a DNA vaccine based on pVAX1, a commercially available plasmid, containing consensus Chinese HIV-1 subtype C *env* gp160 and *gag* genes in one plasmid and *pol* and a *nef/tat* construct designed to express a fusion protein in the second plasmid mixed in a 1∶1 ratio [26], [27] and formulated in sterile isotonic salt solution containing 10 mM sodium phosphate and 150 mM sodium chloride. The 1 mL injection volume corresponded to 4 mg dosage level. TBC-M4 (Lot # 1B,Therion Biologics Corporation, Cambridge MA) is a recombinant MVA virus encoding Indian HIV-1 subtype C *env gp160 (GenBank accession #AF067158), gag (#AF067157), RT (#AF067158), rev (#AF067154), tat (#AF067157)*, and *nef (#AF067154)* genes [4]. The MVA candidate vaccine was formulated in phosphate-buffered saline with 10% glycerol. The respective formulation buffers for each candidate vaccine served as placebos for those candidates. A dose of 0.5 mL of MVA candidate vaccine delivered 5×10^6^ plaque forming units (pfu) (Transgene Biotech company using BHK-21 cell line). The aminoacid sequence homology between the two constructs was more than 85% for most of the proteins (Gag: 95%, Env: 87.1%, Pol/RT: 96.4%), although it was lower for Tat (66.3%) and Nef (18.9%).

### Study Population & Trial Design

This randomized, placebo controlled, double-blind, phase I trial enrolled 32 HIV-uninfected, healthy male and female adult participants from April 2009 to December 2010 at two sites in India: NARI, Pune and NIRT, Chennai.

At the enrolment visit, trial participants were randomized to either Group A or Group B. Participants from Group A received two intramuscular injections of ADVAX (needle administration) or placebo at 0 and 1 months followed by two intramuscular injections of MVA or placebo at 3 and 6 months, while Group B participants received three intramuscular injections of TBC-M4 or placebo at 0, 1 and 6 months. In each group of 16 volunteers, 12 volunteers received trial vaccines and 4 received placebos. At enrollment random allocation was generated by a computer program which was written by EMMES Corporation. The sponsors and study investigators were blinded to the randomization protocol. Volunteers and study staff were not blinded to the vaccine group because of the different numbers of injections in the two groups, but they were blinded to receipt of candidate vaccines or placebos.

### Study Procedures

#### Safety assessment

Local and systemic reactogenicity was assessed on days 0, 3, 7 and 14 after each injection. A physical examination was performed at every visit and protocol-specified laboratory investigations (hematology, biochemistry, immunology and urinalysis) were performed prior to each vaccination, on day 14 after each vaccination and at months 3, 9, 12, and 18 after enrolment. Electrocardiogram (ECG) and plasma cardiac troponin I were assessed at screening and on day 3 after the last vaccination. Adverse events (AE) recorded during the trial were graded using the Division of AIDS (DAIDS, NIAID, NIH) toxicity grading table and the relationship to the study product was assessed as not related, unlikely, possibly, probably or definitely related to the investigational product [28].

#### HIV testing

At screening and at the final study visit, individuals were tested for HIV infection following the algorithm recommended by the National AIDS Control Programme, India [29]. Additionally, HIV testing was performed at each vaccination visit as well as at 3 and 6 months after the last vaccination using HIV ELISA Genetic System and ELAVIA Ac-Ab-Ak1 kits (Bio-Rad Genetic Systems, Marnes-La-Coquette, France). Positive samples by any of the ELISA tests were further tested for HIV viral RNA PCR by Roche Amplicor Version 1.5 (Basel, Switzerland) kit to differentiate vaccine-induced antibodies from antibodies developed subsequent to HIV infection. The difference between the vaccine-induced antibodies and post-HIV infection antibodies was explained to the participants. The volunteers were informed of their seropositivity status at the end of the trial and given a certificate of participation in the trial indicating *this to be a possible reason for continued* seropositivity.

#### Vaccinia antibodies

Anti-vaccinia virus binding antibody titres (VVbAb) were analyzed prior to the first vaccination, 2 weeks after the second vaccination and 2 weeks and 6 months after the last vaccination. VVbAb were tested by V-Bio (St. Louis University, St. Louis, MO) on serum samples using purified vaccinia WR virus as a coating antigen in an ELISA [30]. A positive response to vaccination was defined as baseline titre <100 and post-vaccination titre >100 or baseline titre >100 and post-vaccination titre >2 times the baseline titre.

#### Cellular immunogenicity

Cellular immunogenicity was assessed on the days 0, 7 and 14 after each vaccination as well as at months 3, 9, and 12 after the last vaccination using IFN-γ ELISPOT assay as described previously [4]. The assays were performed on freshly isolated peripheral blood mononuclear cells (PBMCs) at both sites [31]. Briefly, PBMCs were isolated using density gradient separation from heparinised whole blood within 6 hours of blood draw and counted manually or by using a Vi-Cell counter (Beckman Coulter, California, US).The freshly isolated PBMCs were plated at 2×10^5^ per well with synthetic peptides at 2µg/mL.Peptides were 15-mers overlapping by 11 aminoacids, HPLC purified peptides (>90%) encoding the sequences of Gag, Env, Pol, Nef, Tat, Rev for the ADVAX and TBC-M4 vaccine (AnaSpec Inc, Fremont, CA). Negative controls (cells only) and positive controls [cells with 2µg/mL FEC peptides (peptides for Influenza, EBV, Cytomegalovirus) and Phytohemagglutinin at 10µg/mL (PHA, Sigma-Aldrich, St Louis, MO)] were added in each assay. In Group A, cellular immunogenicity was assessed against the ADVAX matched peptides after 1^st^ and 2^nd^ ADVAX vaccinations whereas at enrolment and the later time points both the types of peptides were used. The response in Group B volunteers was assessed using TBC-M4 matched peptides only. The number of spot forming cells (SFC) per 10^6^ PBMCs was counted using an automated ELISPOT reader (AID, Strassberg, Germany). Responses were considered positive based on the criteria determined at the IAVI Human Immunology Laboratory [32] and pre-vaccination responses observed on-site. The results were confirmed at the IAVI Human Immunology Laboratory using frozen PBMCs collected during 4 study visits. A positive response was indicated by: 1) greater than 38–54 SFC/10^6^ cells (depending upon the peptide pool) above the background, 2) more than 4 times the mean background SFC count, 3) less than 70% coefficient variation across the replicate wells and 4) a background <55 SFCs in cell control wells. IFN-γ ELISPOT assays passing validity criteria for positive and negative controls as well as those showing no baseline responses were considered for final analysis.

#### HIV binding antibodies

The HIV-specific antibody responses were evaluated on day 0 and 14 after each vaccination as well as at months 3, 9, and 12 after the last vaccination using commercial ELISA kits mentioned above. The sera from the responders were further tested by HIV-1 Western blot (INNO-LIA™ HIV Score, Innogenetics, Zwijnaarde, Belgium) for assessing the antigen specificity of the HIV antibodies.

#### HIV neutralizing antibodies

Neutralizing antibodies (NAb) were measured as a function of reduction in luciferase reporter gene expression after a single round of infection in TZM-bl cells as described previously [33], [34], [35]. The assays were validated and conducted as a part of the *Collaboration for AIDS Vaccine Discovery (CAVD*). Briefly, heat-inactivated sera from trial participants collected at 14 days and 3 months following last vaccination were incubated with 200 TCID_50_ of pseudoviruses in duplicate in a total volume of 150 µL for 1 hr at 37°C in 96-well flat-bottom culture plates. The panel of *env*-pseudoviruses included Tier-1 and -2 subtype B and C viruses (gifted by the Global HIV Vaccine Research Cryorepository-GHRC, Fraunhofer-Institute, St. Ingbert, Germany) in addition to a CCR5 tropic recently transmitted strain of Indian origin. The viruses used in the panel were SF162.LS- Tier 1 subtype B (cat no. 4694), MW965- Tier-1 subtype C (cat no. 4696), TV1.21- Tier-2 subtype C (cat no. 4659), HIV-001428-2.42- Tier-2 subtype C (cat no. 3551), IVC 4–5 (Tier-2 subtype C: recent infection), MLV- control virus (cat no. 3860).

Freshly trypsinized TZM-bl cells (10,000 cells in 100 µL of growth medium containing 75 µg/mL DEAE dextran) were added to each well keeping appropriate virus and cell controls. After 48 hour incubation, 100 µL of cells was transferred to 96-well black solid plates (Costar, High Wycombe, Bucks, U.K.) for measurements of luminescence using the Britelite Luminescence Reporter Gene Assay System (PerkinElmer Life Sciences, Massachusetts, USA). The results were calculated as the percentage of reduction in Relative Luminescence Units (RLU) in wells containing post-immunization serum relative to the RLU in wells containing corresponding pre-immune serum (collected on the day of vaccination but before vaccination) from the same subject (data not shown). Samples showing more than 50% inhibition were further tested for determination of neutralizing antibody titres using 3-fold dilutions of sera with starting dilution of 1∶10. Nab titers were calculated as the sample dilution conferring a 50% reduction in relative luminescence (ID_50_) as compared to virus control wells after subtraction of background RLU in cell control wells. Validity criteria used for the assays were 1) The average RLU of virus control wells should be >10 times the average RLU of cell control wells, 2) The standard deviation of RLU in the virus control well should be <30%, 3)The standard deviation for duplicate wells should be <30% for sample dilutions that yield at least 40% neutralization, 4) The neutralization curves should be smooth and linear around the 50% neutralization cut-off.

### Statistical Analysis

The sample size of 32 volunteers (*24 vaccine and 8 placebo recipients*) was appropriate for an exploratory clinical trial for evaluating safety while also providing relevant information on vaccine induced immune responses. However, due to the small sample size, the trial had limited power to rule out smaller differences in safety and immunogenicity results between the groups.

All safety and immunogenicity comparisons were made using Fisher’s exact test of the proportions of volunteers with an endpoint, unless otherwise stated. The safety comparisons were based on the maximum severity per volunteer. All comparisons between vaccinated groups are 2-tailed and all comparisons between vaccine and placebo groups are 1-tailed; a significance level of 0.05 was used and due to the exploratory nature of this phase I study no adjustment was made to preserve the overall type I error. The magnitude of responses among the two vaccine groups was compared by the non-parametric Mann Whitney test using GraphPad Prism 5. Analyses were performed using SAS version 9.2, (SAS, Cary, NC, USA).

## Results

### Enrolment and Follow-up

**Figure 1:**
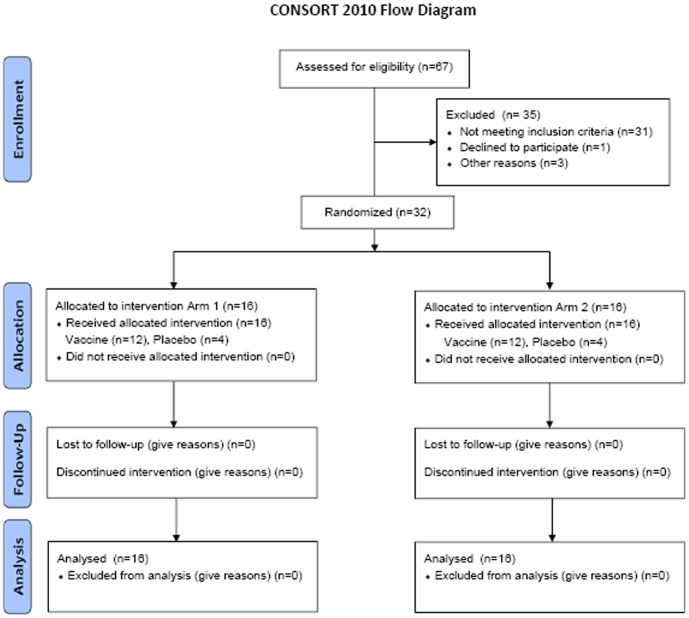
Flow chart of screened and enrolled volunteers.

A total of 32 participants (16 each at NARI and NIRT) were enrolled in the trial between April and June 2009. Groups A and B enrolled 12 vaccine and 4 placebo recipients each. The details of the volunteers screened and enrolled are given in Figure 1. The median age of participants was 34 years with no statistically significant differences between the groups. Overall, 41% of the participants were women, with a male-to-female ratio of 7∶5 in Group A, 6∶6 in Group B and 6∶2 in placebo recipients. All the volunteers were literate. The study follow-up was completed in December 2010 with 100% retention at the last study visit 12 months after the last vaccination.

### Vaccine Safety

After ADVAX vaccinations, 7/12 (58.3%) volunteers in Group A experienced systemic reactogenicity events, graded as mild in 6 volunteers and moderate in one. Three volunteers (25%) in Group A also experienced grade 1 systemic reactogenicity events after MVA vaccinations. Nine of 12 (75%) volunteers from Group B and 5/8 (62.5%) volunteers in the placebo group reported mild systemic reactogenicity. The reported systemic reactogenicity events included malaise, myalgia, subjective fever, nausea, vomiting, arthralgia, fatigue, rash and headache. All except one were mild in severity. Differences in the proportions of volunteers with grade 1 or greater systemic reactogenicity were not statistically significant between the vaccine groups and placebo recipients.

Seven out of 12 (58.3%) volunteers in Group A reported grade 1 local reactogenicity; 5 (41.7%) had reactions after the ADVAX vaccination and 4 (33.3%) after the MVA vaccinations. Nine of 12 (75%) from Group B and 2/8 (25%) receiving placebo demonstrated mild local reactogenicity. Local reactogenicity events included pain and tenderness at the injection site. Differences in the proportions of volunteers with local reactogenicity were not statistically significant between Group A, Group B, and placebo recipients.

**Figure 2:**
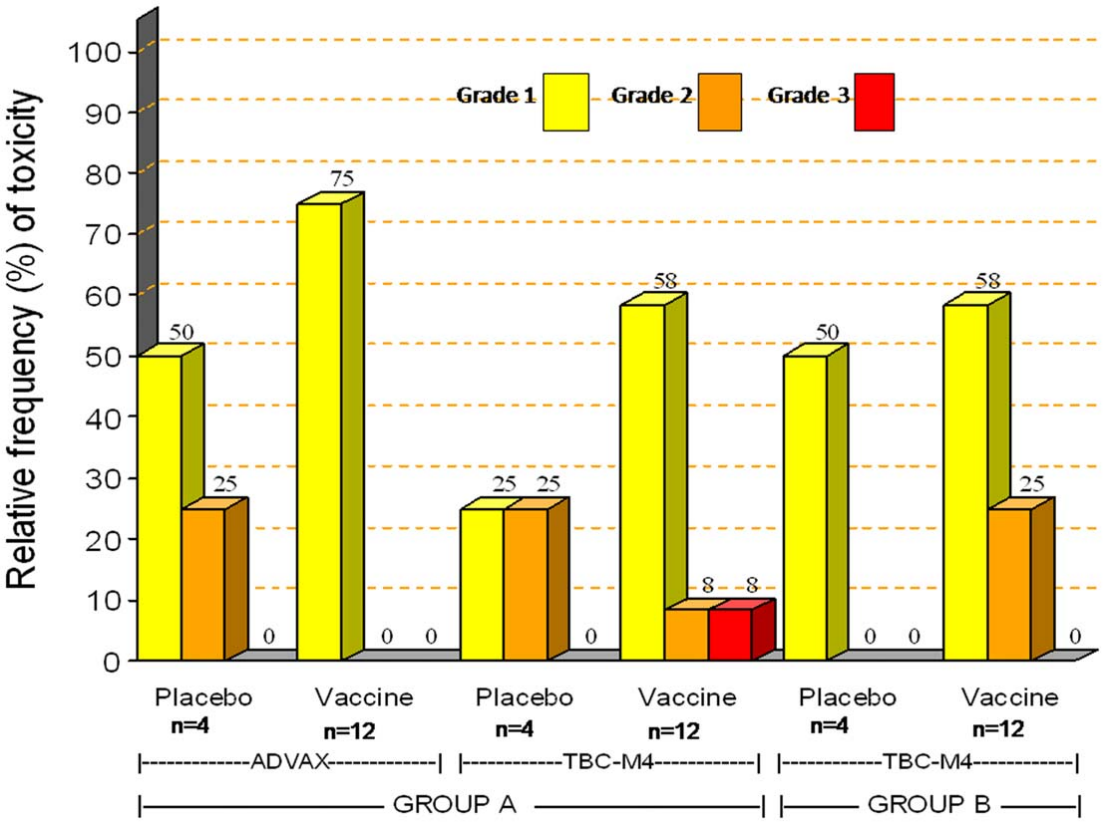
Frequency of grade 1, 2 and 3 unsolicited adverse events recorded within 28 days post-vaccinations. Bars represent the maximum severity per volunteers within 28 days after either 2 vaccinations in Group A (separately for ADVAX and TBC-M4) or 3 vaccinations in Group B. Relative frequency of adverse events is plotted on the Y axis and Groups A, B vaccine or placebo recipients are plotted on the X-axis.

A total of 118 unsolicited adverse events (AE) were reported by 31 of 32 volunteers during the entire study period. Severity and distribution of unsolicited adverse events within 28 days post vaccinations are shown in Figure 2. The distribution of the frequency and severity of AEs was similar in placebo and Group A or B vaccine recipients. None of the adverse events was probably or definitely related to vaccine administration. 103 (87.3%) were assessed as grade 1, 14 (11.8%) as grade 2 and one (0.8%) as grade 3. The latter event was abnormal urinalysis observed in one Group A vaccine recipient (3+ blood, 1+ proteins, 1+ ketones, 2–4 red blood cells/high power field at 12 days after 3^rd^ vaccination). This event resolved without any intervention after one week. Three adverse events in 2 volunteers were assessed as possibly related due to temporal relationship with the vaccinations. These included development of stomatitis 4 days after the second vaccination (Group A), glossitis 1 day post-vaccination (Group B) and premature atrial and ventricular contractions 3 days after the third MVA vaccination (the same Group B volunteer) as detected on ECG, which persisted over 3 months. The volunteer was asymptomatic and his Troponin I levels were within normal limits. This volunteer was referred to a cardiac physician who diagnosed a mild mitral valve prolapse with trivial mitral regurgitation on 2D echo and colour Doppler. The volunteer did not have a past history of any cardiac disease and his earlier ECG done at screening was normal. The volunteer gave history of occasional smoking and alcohol intake. As per the physician, the abnormalities did not translate into clinical symptoms, requiring no further intervention. The ECG abnormalities resolved spontaneously 6 months after the last vaccination without any treatment.

No serious adverse event or death was reported during the study period. One Group A volunteer became pregnant 66 days after the fourth vaccination. This was an unintended pregnancy because of contraceptive failure. The couple was unprepared to bear a child at that time on account of personal problems and the volunteer opted for medical termination of pregnancy.

### Intercurrent HIV Infection

None of the volunteers became HIV-infected during the trial. A summary of the number of volunteers showing positive HIV binding ELISA antibody response is presented in Table 1. All volunteers who showed a positive HIV antibody response at any visit were found to be HIV-uninfected as determined by HIV RNA PCR, ruling out the possibility of acquisition of HIV infection during the study period.

**Table 1:** HIV binding ELISA antibody response rates.

	Group A		Group B		Placebo	
ELISA Response Rate	n	%	N	%	n	%
Post 1st vaccination	0	0	0	0	0/8	0
Post 2nd vaccination	1/12	8	5/12	42	0/8	0
Post 3rd vaccination	7/12	58	12/12	100	0/8	0
Post 4th vaccination	9/12	75	Na	na	0/4	0
3 months post last vaccination	12/12	100	12/12	100	1/8	13
6 months post last vaccination	12/12	100	12/12	100	0/8	0
12 months post last vaccination	4/12	33	7/12	58	0/8	0

### HIV-specific Humoral Immune Responses

**Figure 3:**
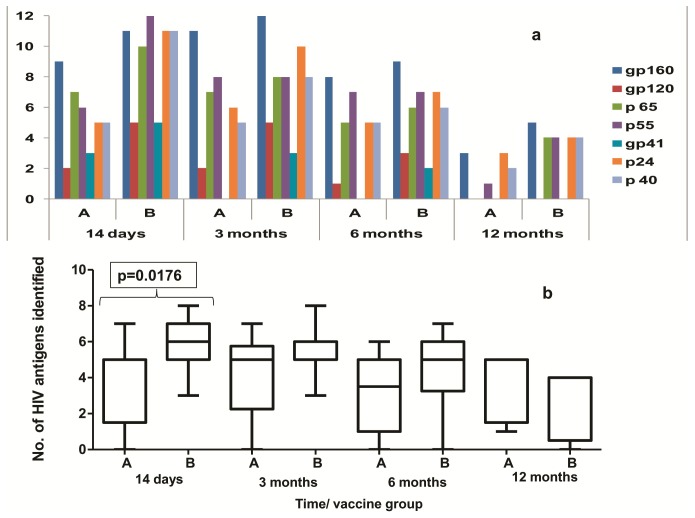
Spectrum of HIV-specific antibodies as determined by Western blot among Groups A and B vaccine recipients. Antigens recognized by HIV-specific antibodies as determined by HIV Western blot assay by group and visit after the last vaccination. Figure 2a shows the frequency of volunteers recognizing each HIV antigen (Env: gp160, gp120 and gp41, Pol: p65, Gag: p55, p24 and p40) by the presence of bands in Western blot. Figure 2b shows the distribution of the spectrum of HIV-specific antibodies (number of HIV antigens identified) by Western blot. median, inter-quartile and minimum-maximum ranges are presented in the Box-Whiskers plots.

None of the volunteers showed the presence of binding antibodies at baseline or after first vaccination. HIV-specific binding antibodies were observed in 58% and 75% of volunteers after the first and second MVA boost in Group A and in 42% and 100% of volunteers after the second and third MVA injections in Group B, respectively. At 3 months after the last vaccination, all volunteers from Group A and Group B showed positive HIV-specific binding antibody responses. However the response persisted in 4 and 7 volunteers from Groups A and B volunteers, respectively at 12 months after the last vaccination (Table 1). The binding antibodies were found to be against Env, Gag and Pol antigens as detected by Western blot (Figure 3a). At 14 days post last vaccination, the breadth of response as defined by recognition of three or more antigens was significantly greater (p = 0.0176) in Group B volunteers. (Figure 3b).

**Figure 4:**
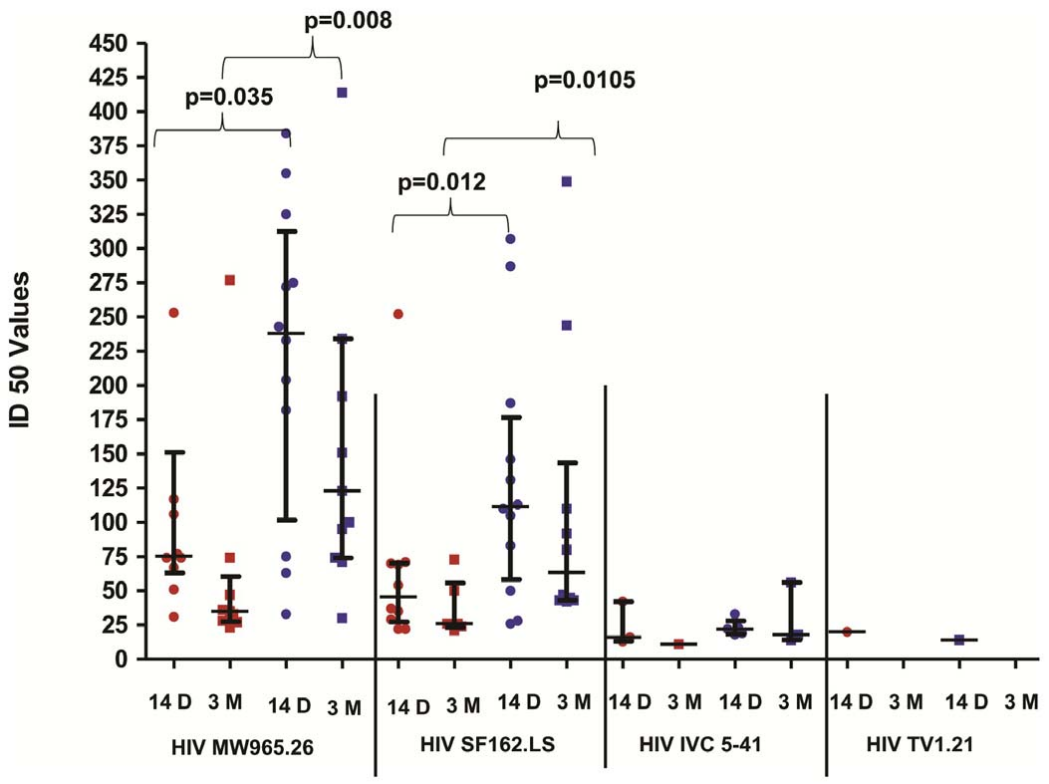
HIV neutralizing antibody titres (expressed as ID_50_ values) of Group A and Group B volunteers after last vaccination. ID_50_ values were determined by TZM-bl assay from serum samples of Group A (red in colour) and Group B (blue in colour) volunteers at 14 days (indicated as circles) and 3 months (indicated as squares) after last vaccination against a panel of pseudoviruses (X-axis). The pseudoviruses shown in the graph are MW965.26 (Tier-1 subtype C), SF162.LS (Tier-1 subtype B), IVC 5–41 (recently transmitted Indian strain) and TV1.21 (Tier-2 subtype C). No neutralization response was seen against HIV 001428-2.42 (Tier-2 subtype C) (not shown). The neutralizing antibody titres from Group B volunteers were found to be higher than in Group A volunteers at 14 days and 3 months following last vaccination. P-values were calculated by the Mann Whitney test. The vertical bars represent median and inter-quartile range.

Fourteen days after the last vaccination, 10/12 vaccinees from Group A and all 12 vaccinees from Group B showed the presence of neutralizing antibodies against Tier-1 subtype C (MW965- median ID_50_ 238 and 76 respectively) as well as subtype B viruses (SF162.LS - median ID_50_ 112 and 46 respectively). The neutralizing antibody titres were higher in Group B than in Group A volunteers (p<0.05) (Figure 4). However the titres decreased at 3 months after the last vaccination. Interestingly, 3/10 from Group A and 5/10 from Group B volunteers showed neutralizing activity against IVC 5–41,a recently transmitted strain of Indian origin, although the titres were low (range: 11–42). No neutralizing activity was detected against Tier-2 viruses except for the positive response against TV 21 shown by one participant in Group A. None of the placebo recipients showed presence of HIV neutralizing antibodies and neutralizing activity was not observed against control virus (SVA-MLV).

### HIV-specific Cellular Immune Responses

Overall 398 out of a total of 423 IFN-γ ELISPOT assays performed during the trial period were found to be valid. Table 2 describes responses to any peptide within 2 weeks of each vaccination in case of both TBC-M4 matched and ADVAX matched peptides. In Group A, positive responses were infrequent following DNA vaccination, with only 3/12 volunteers showing a response after the second DNA vaccination. However, all 12 Group A vaccinees responded to both TBC-M4 and ADVAX-matched peptides after both the first and second MVA vaccinations. In Group B, 6/10, 6/11 and 11/12 volunteers showed positive responses after the first, second and third MVA vaccinations, respectively. TBC-M4-matched peptide responses persisted up to 3, 6 and 12 months post-last vaccination in 8/11, 8/12 and 3/10 volunteers from Group A, and in 7/12, 4/12 and 4/11 volunteers from Group B respectively. In placebo recipients, a few positive responses against either ADVAX or TBC-M4 peptides were detected after each vaccination giving over all false positive rate of 3.6%, which is comparable to what has been reported by IAVI (4.1%) and HVTN (5.1%) laboratories [32].

**Table 2:** Frequency of IFN- γ ELISPOT T-cell response.

	Group A ADVAX-matched peptide		Group A TBC-M4-matched peptide		Group B TBC-M4-matched peptide		Placebo Any Peptide	
Overall Response Rate	n	%	n	%	n	%	n	%
Post 1^st^ Vaccination	0/12	0.0	nd	Nd	6/10	60.0	1/8	12.5
Post 2^nd^ Vaccination	3/12	25.0	nd	Nd	6/11	54.5	2/8	25.0
Post 3^rd^ Vaccination	12/12	100.0	12/12	100.0	11/12	91.7	3/8	37.5
Post 4^th^ Vaccination	12/12	100.0	12/12	100.0	na	na	2/4	50.0
3 months post vaccination	10/11	90.9	8/11	72.7	7/12	58.3	1/7	14.3
6 months post last vaccination	9/12	75	8/12	66.7	4/12	33.3	0/7	0
12 months post last vaccination	4/10	40	3/10	30	4/11	36.40	0/6	0

na: Not Applicable, nd: not done.

**Figure 5:**
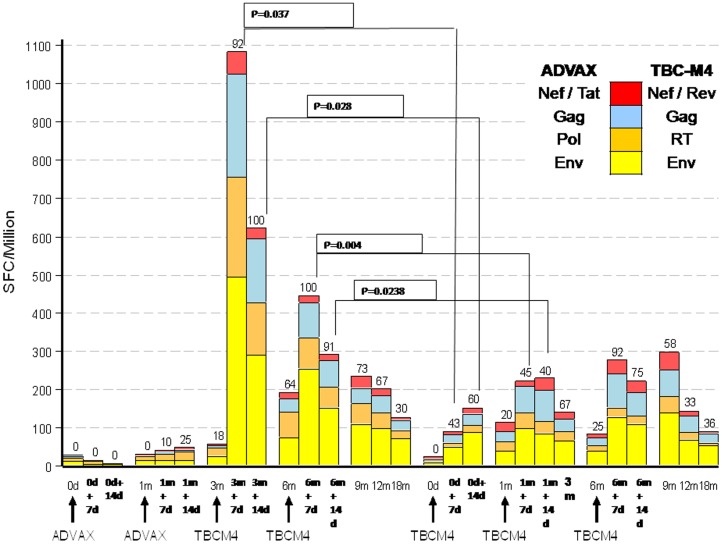
T-cell immune responses as assessed by IFN-γ secretory ELISPOT assays against ADVAX matched peptides after 1^st^ and 2^nd^ DNA vaccinations in group A and TBC-M4 matched peptides at the other time points in group A and at all the time points in group B participants are shown. Average magnitude of IFN-γ ELISPOT responses in SFC/10^6^ cells (Y-axis) at each time point, by Groups A and B volunteers (X-axis) against different antigens are represented by different colours. 7d and 14d indicate 7 and 14 days after every vaccination, respectively. Values above the bars represent the percent of volunteers with positive responses to any or at least one peptide at that visit. The black lines and the corresponding p values showed comparison between the responses in group A and group B at days 7 and 14 after the first and second MVA vaccinations.

The proportion of volunteers with at least one response to a TBC-M4-matched peptide was significantly (p<0.05) greater in group A than Group B at day 7 and 14 after the first and second MVA vaccinations as shown in Figure 5 indicating the priming effect of the DNA vaccination. However the proportion of Group A and Group B responders at 9, 12 and 18 months (corresponding to 3, 6 and 12 months post last vaccination) were not different at a statistically significant level. The ELISPOT responses were predominantly seen against Env (11 in Group A and 9 in Group B) and Gag (8 in Group A and 11 in Group B) peptides followed by Pol (7 in Group A and 3 in Group B). Responses against Nef/Rev/Tat peptides were infrequent (2 in Group A and 3 in Group B). In group A, the magnitude of TBC-M4-matched peptide responses was found to be highest at 7 days following the first MVA boost however it was found to be lower after the second MVA boost. The distribution of the magnitude of IFN-γ ELISPOT responses in Group B was similar after the second and third vaccinations, with minor boosting observed after the first vaccination. The breadth (recognition of one or more HIV antigens) of TBC-M4-matched peptide responses was greater in group A than in group B, but the difference (based on Wilcoxon’s Rank Sum test for the breadth of response per volunteer) was not statistically significant, except on day 14 following the first MVA vaccination (p = 0.024, unadjusted for multiple comparisons).

### Vaccinia ELISA Binding Antibody

Vaccinia ELISA binding antibody titres (VVbAb) were detected at baseline in 3/12, 4/12 and 1/4 volunteers in Groups A, B and placebo recipients, respectively. All vaccinated volunteers from both groups had a positive vaccinia binding antibody titre after MVA vaccination. None of the placebo recipients showed an increase in anti-vaccinia antibody titres over the baseline titre. At 6 months post-final vaccination the median titres were lower (418 and 616 in Group A and B, respectively) compared to 14 days post final vaccination (1442 and 946 in Groups A and B, respectively) (Data not shown).

## Discussion

This phase I clinical trial was conducted with the objective of comparing safety and immunogenicity of the heterologous DNA/MVA with homologous MVA alone regimens. Several studies have shown that DNA priming improves the quality of both T-cell and B-cell immune responses, when boosted with viral vectors [13], [14], [15]. Hence, a heterologous DNA prime and MVA boost vaccination regimen was designed to investigate whether a more robust immune response would be generated in comparison with a homologous MVA boost regimen. However, due to the small sample size, this study had limited power for analytical comparisons of safety and immunogenicity responses between the groups receiving two types of regimens.

Both vaccine strategies were shown to be generally safe and well tolerated. No vaccine-related serious adverse events were observed. Volunteers experienced mostly mild local and systemic reactogenicity. The overall distribution of local and systemic reactogenicity, as well as unsolicited adverse events, was similar among vaccine and placebo recipients. In the present study, one Group B volunteer showed premature atrial and ventricular contractions which were detected at 3 days post third MVA injection. The ECG abnormality resolved spontaneously without any treatment. MVA, being a non replicating vector, has not been previously reported to cause pericardiac or myocardiac events [19], [36]. Although the condition was categorized as possibly related in view of the temporal relationship of the ECG abnormality and the known rare risk of myocarditis following replicating vaccinia vaccination, the event did not meet the criteria of pericarditis or myocarditis, and thus does not point to an association between ECG abnormality and TBC-M4 (a non-replicating vector) administration. The episode of glossitis observed in the same volunteer could be due to an inter-current enterovirus infection for which unfortunately no specific serology could be performed.

Immunogenicity assessments after the study vaccinations demonstrated sporadic immune responses after the DNA vaccinations in Group A. This result was expected since DNA vaccines administered by needle injection, in the absence of an adjuvant, are generally weakly immunogenic. The priming effect of DNA was evidenced after the first MVA vaccination in Group A, where 100% of subjects showed a positive IFN-γ ELISPOT response, compared to 60% of subjects in Group B after the first MVA vaccination. This finding suggests that DNA vaccination did prime the immune system as has been reported earlier [15]. The mechanism by which DNA priming exerts such effects remains to be elucidated. One of the suggested mechanisms is induction of HIV-specific CD4+T-cells which might help in rapid expansion of CD4+ and CD8+T-cell responses during boosting [13], [36]. Although CD4+ T cells are also implicated in long term persistence of immune response, no difference in persistence of IFN-γ ELISPOT response was observed at 12 months after the last vaccination in the present study.

Both the DNA and MVA constructs contained multiple genes of HIV with the intent of targeting several viral components. This strategy is thought to be useful for reducing the risk of escape from vaccine-induced immunity [37], [38]. Prime-boost immunization has been shown to significantly increase the breadth of the immune responses possibly due to the divergent cell targeting and antigen processing routes complementing one another, allowing a greater diversity of epitope recognition than with either agent alone [39]. However, in the present study, the breadth of T-cell responses as defined by recognition of multiple HIV antigens was similar in both groups. The T-cell responses were predominantly seen against Env and Gag as observed in previous studies [40]. Predominant Env specific responses have also been reported after vaccinations with multi-genic poxvirus vectors previously [41], [42]. Although in natural chronic HIV infection, Env-specific CD8+ T-cell responses have been shown to be associated with poor control of viral replication compared to Gag-specific responses [43], non-human primates immunized with DNA plus Ad5 expressing SIV Env as well as Gag were better protected against SIV challenge compared to animals immunized with vaccines expressing only Gag [44]. Another macaque study also showed that the protection against the acquisition of SIV infection required the inclusion of Env in the vaccine regimen [45] indicating that the generation of Env-specific responses might also be important in early HIV infection.

The MVA-alone strategy was evaluated earlier in a previous clinical trial conducted in India [4], where the low dose used was comparable in titer to the MVA dose used for vaccination in the present study. The response rates in the current Group B are comparable to those reported in the low dose group of the previous trial, with 64% and 91% of volunteers showing positive T-cell responses in the previous trial as compared to 55% and 92% after the second and third MVA vaccinations, respectively, in the current trial. Among MVA vaccine recipients in both trials, the response magnitudes were modest and directed against Env and Gag.

In the present study, antibody responses, as opposed to T-cell responses, were of higher frequency, magnitude and breadth of recognized HIV antigens by Western blot in the MVA only group (Group B) than in the DNA/MVA group (Group A). This corroborates a macaque study using MVA alone that showed 10-fold higher anti-Env antibodies following immunization with MVA alone compared to those immunized with DNA and MVA regimens [46]. Conversely, in some studies the antibody responses to prime-boost regimens with DNA as prime and Ad5 or protein as boost, were found to be higher than those in homologous Ad5 or protein boost regimens [15]. One of the reasons for this could be the ability of poxviruses to act as adjuvants for B cells through induction of TNF-α and IL-6, supporting plasma cell survival [47]. By contrast, T-cell responses failed to increase in magnitude after repeated boosting with MVA in both the groups. Similar results with failure of boosting of the T-cell response with a marginal increase in antibody responses to HIV proteins after repeated immunizations of MVA have also been observed previously in animal models [46], [48]. Failure to boost the T-cell immune response against vector expressed foreign antigen after repeated immunizations may be due to induction of immune responses against viral vector proteins leading to early elimination of vectors hampering immune responses against the vaccine insert. Despite the differences observed in immune responses elicited by DNA prime MVA boost compared with homologous MVA boost regimens, the protection against SIV challenge in macaques has been shown to be comparable in one of the previous study [49].

Vaccinia antibodies were induced in all vaccinated volunteers after MVA vaccination in both groups. All the 8 volunteers with baseline responses were born before 1980 and hence the antibodies are likely to have been induced by a previous smallpox vaccination. No correlation was detected between the magnitude of IFN-γ ELISPOT response with the presence of VVbAbs at baseline (data not shown) suggesting that pre-existing immunity did not influence the induction of HIV-specific immune responses in these volunteers, as reported earlier [4], [50]. This is in contrast with data showing high baseline adenovirus type 5-specific antibody titres and hampering IFN-γELISPOT responses [51].

Titres of HIV-specific neutralizing antibodies were significantly higher in the MVA only group (Group B) volunteers as compared to the DNA/MVA group (Group A). The neutralizing antibody response was mainly against Tier-1 subtype B and subtype C viruses, which are known to be neutralization-sensitive. No responses against Tier-2 viruses were detected in any volunteers. It has already been shown that Tier 2 responses are rarely induced by most of the vaccine strategies tested so far in clinical trials [52], [53]. Interestingly, 3/12 and 5/12 volunteers from Groups A and B, respectively, showed presence of neutralizing antibodies against a recently transmitted strain of Indian origin. This could be considered as an important determinant of vaccine-induced immunity indicative of possible protection against transmitted HIV. Furthermore, although neutralizing antibodies against Tier-2 viruses were minimal, the role of non-neutralizing antibodies in protecting against HIV cannot be ruled out, as emphasized in the RV144 trial, where protection was found to be associated with presence of anti V1/V2 binding IgG antibodies [54], [55].

In conclusion the safety profile as well as immunogenicity of the DNA/MVA heterologous prime-boost strategy was comparable with that of the homologous MVA alone strategy. Although DNA priming resulted in enhancement of immune responses following 1^st^ MVA boosting in group A, the effect lasted for a very short time demonstrating no immunological advantage of heterologous prime boost strategy over homologous MVA alone strategy. New administration strategies of DNA vaccines to augment T-cell immune responses including intra-dermal needle injection, Biojector, and electroporation with or without molecular adjuvant administration have generated promising results that may deserve further investigations with vector boosts [56], [57], [58].

## Supporting Information

Protocol S1 **Trial Protocol.**
(PDF)

Checklist S1 **CONSORT Checklist.**
(PDF)
