# Supplementary material for: Safety and Immunogenicity of DNA and MVA HIV-1 Subtype C Vaccine Prime-Boost Regimens: A Phase I Randomised Trial in HIV-Uninfected Indian Volunteers
Source: PLoS One. 2013 Feb 13;8(2):e55831. doi: 10.1371/journal.pone.0055831 (PMC3572184; doi:10.1371/journal.pone.0055831)
Supplement: Protocol S1 — Trial Protocol. (PDF) [file pone.0055831.s001.pdf]

Ethics Committee
NATIONAL AIDS RESEARCH INSTITUTE
(INDIAN COUNCIL OF MEDICAL RESEARCH)
Plot No. 73, 'G' Block, MIDC, Bhosari, Pune 411026

Date: 16 May 2008

To
Dr. S. M. Mehendale
Deputy Director (SG) & Scientist F
NARI

Sub: Approval of Protocol No. IAVI P001 Phase I Study and Study Documents.

Reference: Your letter dated 28 April 2008, NARI/IAVIP001/08-39

Study: A Phase I Double Blind, Placebo Controlled, Randomized Trial to evaluate the Safety and Immunogenicity of TBC-M4, a multigenic MVA HIV Vaccine versus ADVAX, a multigenic DNA HIV Vaccine followed by TBC-M4, a multigenic MVA HIV Vaccine

Protocol Version: 1.0 dated 11th March 2008.

Principal investigator: Dr. S.M. Mehendale

Dear Dr. S. M. Mehendale

The above-mentioned study and related documents were reviewed in the Ethics Committee Meeting held on 14th May 2008. The Ethics Committee hereby approves the following documents:

1. Study Protocol, version 1.0, dated 11th March 2008.
2. Informed Consent documents in English and Marathi language of the following:
- P001 Phase I Protocol Screening Informed Consent Document (English and Marathi) version 1.0, 18 April 2008.
- P001 Phase I Protocol Enrollment Informed Consent Document (English and Marathi) version 1.0, 18 April 2008.
- P001 Phase I Protocol Volunteer's Information Brochure (English and Marathi) version 1.0, 18 April 2008.
- Volunteer's Assessment of understanding (English and Marathi) version 1.0, 18 April 2008.
3. Infokits – Information for potential volunteers in English and Marathi languages of the following Infokit version 1.0, 18 April 2008:
- Frequently Asked Questions on HIV/AIDS (English and Marathi)
- Frequently Asked Questions on AIDS vaccine (English and Marathi)

- Getting to know the trial organizers (English and Marathi)
- A message of motivation (English and Marathi)

The committee also acknowledges the receipt of the following Investigators Brochure documents:

1. ADVAX e/g + ADVAX p/n-t Subtype C- based env/gag and pol/nef-tat Plasmid DNA Vaccine → Edition 2.0 May 2007
2. TBC-M4 : MVA HIV-1 Multigenic Subtype C Vaccine → Version 2.0-17 December 2007

This approval is valid up to 13 May 2009.

Regards

Dr. R.K. Mutatkar
Chairman, Ethics Committee
NARI

Trial Protocol

ADVAX and TBC-M4 Prime-Boost Trial

**Protocol Title**	A Phase I Double-Blind, Placebo-Controlled, Randomized Trial to Evaluate the Safety and Immunogenicity of TBC-M4, a multigenic MVA HIV Vaccine vs ADVAX, a multigenic DNA HIV Vaccine followed by TBC-M4, a multigenic MVA HIV Vaccine
**Protocol Number**	IAVI P001
**Phase**	Phase I
**Sponsor**	International AIDS Vaccine Initiative (IAVI)<br>110 William Street, 27th Floor<br>New York, New York 10038-3901<br>USA
**Study Sites**	Tuberculosis Research Centre<br>Mayor VR Ramanathan Road<br>Chetpet, Chennai 600031<br>India<br><br>National AIDS Research Institute<br>Plot # 73, Block G<br>MIDC Bhosari, Pune, 411 026<br>India
**Date of Protocol**	11 March 2008
**Version**	1.0

The **CONFIDENTIAL INFORMATION** in this document is provided to you as an Investigator, potential Investigator, or Consultant, for review by you, your staff, and applicable institutional review and independent ethics committees. It is understood that the information will not be disclosed to others, except to the extent necessary to obtain ethical and regulatory approval from the respective committee's agencies and informed consent from those persons to whom the investigational product may be administered.

Synopsis

**Title**	A Phase I Double-Blind, Placebo-Controlled, Randomized Trial to Evaluate the Safety and Immunogenicity of TBC-M4, a multigenic MVA HIV Vaccine vs ADVAX, a multigenic DNA HIV Vaccine followed by TBC-M4, a multigenic MVA HIV Vaccine
**Protocol Number**	IAVI P001
**Phase**	Phase I
**Sponsor**	International AIDS Vaccine Initiative (IAVI)<br>110 William Street, 27th Floor<br>New York, New York 10038-3901<br>USA
**Study Sites**	Tuberculosis Research Centre<br>Mayor VR Ramanathan Road<br>Chetpet, Chennai 600031<br>India<br><br>National AIDS Research Institute<br>Plot # 73, Block G<br>MIDC Bhosari, Pune, 411 026<br>India
**Collaborating Companies:**	Aaron Diamond AIDS Research Center<br>455 First Avenue<br>New York, NY 10016, USA

Objectives

Primary
To evaluate the safety of intramuscularly administered ADVAX DNA HIV vaccine at time 0 and 1 month, followed by TBC-M4 at months 3 and 6, compared with TBC-M4 administered intramuscularly at 0, 1, and 6 months.

Secondary
To evaluate the immunogenicity of intramuscularly administered ADVAX DNA HIV vaccine at time 0 and 1 month, followed by TBC-M4 at months 3 and 6, compared with TBC-M4 administered intramuscularly at 0, 1, and 6 months.

Other
To examine the safety and immunogenicity of the construct in the presence of pre-existing immunity against vaccinia, if any participants have such immunity
.

Study Design

Group	N (vaccine/placebo)	Month			
		0	1	3	6
A	12/4	ADVAX	ADVAX	TBC-M4	TBC-M4
B	12/4	TBC-M4	TBC-M4		TBC-M4

Study population

Healthy male or female adults, 18-50 years of age, who are not infected with HIV-1 or HIV-2; who do not have reported high-risk behavior for HIV infection; who are available for the duration of the trial, who are willing to undergo HIV testing, use an effective method of contraception, and who, in the opinion of the principal investigator or designee, understand the study and can provide written informed consent.

Principal exclusion criteria include: HIV infection; pregnancy and lactation; a chronic disease which in the opinion of the investigators makes the volunteer unsuitable for the trial; clinically significant abnormal laboratory values, recent vaccination or receipt of a blood product or experimental agent; and previous severe vaccine reaction or having previously received an HIV vaccine candidate.

Clinical sites	National AIDS Research Institute (NARI), Pune, India<br>Tuberculosis Research Centre (TRC), Chennai, India
Clinical laboratory	TRC Laboratory, Chennai, India<br>NARI Laboratory, Pune, India
Immunological laboratories	TRC Laboratory, Chennai, India<br>NARI Laboratory, Pune, India<br>IAVI Core Laboratory, Imperial College, London, UK
Number of volunteers	32 volunteers, 24 receiving vaccine and 8 placebo recipients. Two groups to be enrolled at TRC, Chennai and NARI, Pune

The clinical trial protocol will be submitted by each study site to their respective Scientific and Ethics Committees. The clinical trial will begin once all approvals have been obtained from the above-mentioned committees, ICMR and national regulatory bodies in India including the Drug Controller General of India, Genetic Engineering Approval Committee and Health Ministry's Screening Committee or Department of Health Research,

Description of the Investigational Product

The study vaccines are as follows:

- ADVAX consists of one plasmid containing Clade C HIV *env* and *gag* and one plasmid containing Clade C HIV *pol* and *nef/tat* fusion mixed in a 1:1 ratio.
- TBC-M4, an attenuated form of modified vaccinia Ankara (MVA) virus vector, contains *env*, *gag*, *tat-rev*, and *nef-RT* HIV-1 subtype C genes.

The associated placebos are as follows:

1. The ADVAX placebo is the formulation buffer, which is a sterile phosphate saline buffer, composed of 0.01M sodium phosphate, 150mM NaCl, pH 7.2.
2. The TBC-M4 placebo is the formulation buffer, which is a sterile 0.01M Phosphate Buffered Saline (150mM NaCl) buffer pH 7.0 containing 10% glycerol.

Vaccine/ Placebo	Dosage Level	Total Injected Volume	Route of Administration
ADVAX	4 mg	1 mL	IM
ADVAX Placebo	NA	1 mL	IM
TBC-M4	$5x10^6$ pfu	0.5 mL	IM
TBC-M4 placebo	NA	0.5 mL	IM

IM=Intramuscular

Blinding
Study site staff and volunteers will not be blinded with respect to group assignment (e.g., group A or B) but will be blinded to the allocation of the product (vaccine or placebo). The placebo to be used in each group will be the formulation buffer of each vaccine.

Duration of study participation
Volunteers in all groups will be followed for 12 months after receiving their last vaccination.

Primary endpoints

Safety

- Proportion of volunteers with Grade 3 or 4 local reactogenicity events (injection site pain, tenderness, pruritis, cutaneous reaction, erythema, induration)
- Proportion of volunteers with Grade 3 or 4 systemic reactogenicity events (fever, fatigue, malaise, chills, pain, nausea, vomiting, myalgia, arthralgia)
- Proportion of volunteers with other Grade 3 or 4 severity adverse events (AEs) (including laboratory abnormalities) and with AEs considered related to vaccine.
- Proportion of volunteers with Serious Adverse Events (SAEs) and with SAEs considered related to vaccine.

Secondary endpoints

Safety and tolerability

- Proportion of volunteers with other adverse events.

Immunogenicity

- Proportion of volunteers with HIV-1 specific T-cell responses by ELISPOT assay (if robust responses occur, they will be characterized by flow cytometry for intracellular cytokines and surface markers such as CD4 and CD8)
- Proportion of volunteers with antibodies to HIV antigens
- Proportion of volunteers with binding antibodies to vaccinia
- Proportion of volunteers showing *in vitro* viral inhibition[1]

Evaluation for intercurrent HIV infection
Volunteers will be tested for HIV-1 and HIV-2 antibodies according to the Schedule of Procedures or if medical or social circumstances dictate, using rapid tests and ELISA. Should one or both of the routine post-vaccination test(s) be positive, a pre-defined testing algorithm will be followed to determine whether antibodies have been induced by the vaccine or whether the volunteer has become infected with HIV-1 through exposure in the community. Results will be reported to the clinical team as “HIV-infected” or “not HIV-infected” to prevent unblinding of the staff and suitably explained to the volunteers.

Statistical considerations
Data will be recorded on the Case Report Form (CRF) and will be available to designated study staff in blinded form. An analysis of grouped data will be carried out without unblinding the study to investigators or volunteers when half the volunteers have completed their vaccination schedule (see Section 5.2.). At the end of the study, a full analysis will be prepared according to a pre-specified data analysis plan.

Safety and tolerability will be assessed by examining the overall rates of Grade 1-4 and serious adverse events and unusual or unexpected events that might be associated with vaccination and the number of volunteers who experience these events. All clinical and routine laboratory data will be included in the safety analysis.

ABBREVIATIONS

AE	Adverse event
ADARC	Aaron Diamond AIDS Research Center
AIDS	Acquired human immunodeficiency syndrome
ALT	Alanine-aminotransferase
ANC	Absolute neutrophil count
ALC	Absolute lymphocyte count
AST	Aspartate-aminotransferase
CCID50	Cell Culture Infectious Dose 50%
CD 4, CD8	T lymphocytes of cluster differentiation 4 and 8
CDC	Center for Disease Control, Atlanta, USA
CFC	Cytokine flow cytometry
CRF	Case Report Form
CTL	Cytotoxic T lymphocyte
DCC	Data Coordinating Centre
DNA	Desoxyribonucleic acid
DRP	DNA resistant particle
DSMB	Data Safety Monitoring Board
ECG	Electrocardiogram
ELISA	Enzyme Linked Immunosorbent Assay
ELISPOT	Enzyme Linked Immunosorbent Spot Assay
ET	Early Termination
GCP	Good Clinical Practice
GLP	Good Laboratory Practice
HBV	Hepatitis B Virus
HCV	Hepatitis C Virus
HIV	Human Immunodeficiency Virus
IAVI	International AIDS Vaccine Initiative
ICH	International Conference on Harmonisation
ICMR	Indian Council of Medical Research
IUCD	Intrauterine Contraceptive Device
KAVI	Kenya AIDS Vaccine Initiative
MCA	Medicines Control Agency
MVA	Modified Vaccinia Ankara
NA	Not applicable
NACO	National AIDS Control Organization, India
NARI	National AIDS Research Institute, Pune, India
NEC	National Ethics Committee
PBMC	Peripheral blood mononuclear cells
PBS	Plasma buffer solution
PI	Principal Investigator
PCR	Polymerase chain reaction
PFU	Plaque forming unit
RPR	Rapid Plasma Reagin
RT	Reverse transcriptase
SAE	Serious Adverse Event
SIV	Simian immunodeficiency virus
SOP	Standard Operating Procedure
SRSV	Small round structure virus
TCID50	Tissue Culture Infectious Dose 50%
TPHA	*Treponema pallidum* hemagglutination
TRC	Tuberculosis Research Centre
TWG	Trial working group
UK	United Kingdom
ULN	Upper Limit of Normal
USA	United States of America
V.V.	Vaccinia virus

TABLE OF CONTENTS

ABBREVIATIONS 6
1.0 SPONSOR CONTACT INFORMATION........ 9
2.0 SIGNATURE PAGE........ 12
3.0 BACKGROUND INFORMATION........ 14
3.1. Study Rationale 15
4.0 STUDY OBJECTIVES 18
4.1 Primary Objectives........ 18
5.0 STUDY ENDPOINTS AND STUDY DESIGN........ 18
5.1 Study Endpoints........ 18
5.1.1 Primary endpoints 18
5.1.2 Secondary endpoints 19
5.2 Study Design 19
5.2.1 Duration of study........ 19
5.2.2 Study population 20
5.2.3 Inclusion criteria 20
5.2.4 Exclusion criteria........ 20
5.2.5 Recruitment of study volunteers........ 22
6.0 STUDY VISITS........ 22
6.1 Screening 22
6.2 Vaccination Visit 23
6.3 Post-Vaccination Follow-Up Visits 24
6.4 Additional Follow-Up Visits........ 24
6.5 Final Study Visit and Early Termination Visit........ 24
7.0 INVESTIGATIONAL PRODUCTS........ 24
7.1 Description of ADVAX and placebo 25
7.1.1 Active vaccine 25
7.1.2 Placebo........ 25
7.2 Description of TBC-M4 Study Vaccine and Placebo 25
7.2.1 Active vaccine........ 25
7.2.2 Placebo........ 25
7.3 Shipment........ 26
7.4 Storage 26
7.4.1 Storage of ADVAX........ 26
7.4.2 Storage of TBC-M4........ 26
7.5 Dispensing and Handling........ 26
7.5.1 Dispensing and Handling of ADVAX 26
7.5.2 Dispensing and Handling of TBC-M4........ 26
7.6 Administration of Investigational Products 26
7.7 Accountability and Disposal........ 26
8.0 STUDY PROCEDURES 27
8.1 Informed Consent 27
8.2 Medical History and Physical Examination........ 28
8.3 HIV Risk Assessment, HIV Testing and HIV Test Counseling 28
8.4 Family Planning Counseling 28
8.5 Blood Collection and Shipment........ 28
8.6 Compensation for Participation........ 28
8.7 Randomization and Blinding 29
8.8 Unblinding Procedure 29
9.0 ASSESSMENT........ 29
9.1 Safety Assessment........ 29
9.1.1 Local reactogenicity events........ 29
9.1.2 Systemic reactogenicity events........ 29
9.1.3 Other adverse events........ 30
9.1.4 Concomitant Medications........ 30
9.1.5 Routine laboratory parameters........ 30
9.1.6 Specific screening tests 31
9.1.7 Cardiac Assessments 31
9.1.8 Double Stranded DNA Antibody tests........ 32

9.1.9 Psychological Assessment 31
9.2 Immunogenicity Assessments 31
9.2.1 HIV Antibody responses 31
9.2.2 Cellular responses 32
9.2.3 Other Immunological Assessments 32
9.3 PBMC, Serum and Plasma Storage 32
9.4 Other Assessments 32
9.4.1 HLA typing 32
9.4.2 HIV test 32
9.4.3 Antibody response to the MVA vector 33
9.4.4 Viral Inhibition Assays 33
9.4.5 Pregnancy test 33
9.4.6 Concomitant medication 33
10.0 ADVERSE EVENTS 33
10.1 Definitions 33
10.2 Assessment of Severity of Adverse Events 33
10.3 Relationship to Investigational Product 33
10.4 Serious Adverse Events 34
10.5 Clinical Management of Adverse Events 35
10.6 Pregnancy 35
10.7 Intercurrent HIV Infection 36
11.0 MANAGEMENT OF HIV ISSUES DURING AND FOLLOWING THE TRIAL 36
11.1 HIV Risk Assessment, HIV Testing and HIV Test Counseling 36
11.2 HIV infection 36
11.2.1 Counselling 37
11.2.2 Referral for support and care 37
11.3 Social Discrimination as a Result of an Antibody Response to Investigational Product 37
12.0 DISCONTINUATION OF VACCINATIONS AND/OR WITHDRAWAL FROM THE STUDY 37
12.1 Discontinuation of Vaccinations 37
12.2 Follow-up after discontinuation of vaccinations 38
12.3 Withdrawal from the Study (Early Termination) 39
12.4. Follow-up Withdrawal from the Study (Early Termination) 39
13.0 DATA HANDLING 39
13.1 Data Collection and Record Keeping at the Study Site 39
13.2 Data Collection and Transfer at the IAVI Core Laboratory 39
13.3 Data Entry at the Study Site 40
13.4 Data Analysis 40
14.0 STATISTICAL CONSIDERATIONS 40
14.1 Sample Size 40
14.2 Statistical Power and Analysis 40
15.0 QUALITY CONTROL AND QUALITY ASSURANCE 42
16.0 DATA AND BIOLOGICAL MATERIAL 42
17.0 ADMINISTRATIVE STRUCTURE 42
17.1 Data Safety Monitoring Board (DSMB) 42
17.1.1 Content of Interim Review 42
17.1.2 Indications for Discontinuation of Vaccinations in all Volunteers 43
17.2 Study Supervision 43
18.0 INDEMNITY 43
19.0 PUBLICATION AND COMMUNICATION 43
20.0 ETHICAL CONSIDERATIONS 43
APPENDIX A - SCHEDULE OF PROCEDURES 44
APPENDIX B - ADVERSE EVENT SEVERITY ASSESSMENT TABLE 46
REFERENCES 58

1.0 SPONSOR CONTACT INFORMATION

**Study Sponsor**		
International AIDS Vaccine Initiative (IAVI)	Telephone:	+ 1 212 847 1111
110 William Street, 27th Floor	Fax:	+ 1 212 847 1113
New York, NY 10038-3901 USA		
**Study Chairs**		
Dr. Ramesh Paranjape	Telephone	+ 91 20 2712 1342
Scientist G & Director	Fax:	+ 91 20 2712 1071
National AIDS Research Institute	Mobile:	+ 91 94220 88135
Plot # 73, Block G,	E-mail:	rparanjape@nariindia.org
MIDC Bhosari, Pune, 411 026, India		
Dr. PR. Narayanan	Telephone:	+91 44 2836 9600
Scientist G & Director	Fax:	+ 91 44 2836 2528
Tuberculosis Research Centre	Mobile:	+ 91 9444 058568
Chetpet, Chennai, 600 031, India	E-mail:	prnarayanan@trcchennai.in
**Principal Investigator (NARI)**		
S.M Mehendale, MBBS, MD, MPH	Telephone:	+ 91 20 2712 1342
Scientist F,	Fax:	+ 91 20 2712 1071
Division of epidemiology and	Mobile:	+ 91 94220 87979
Biostatisics	E-Mail:	smehendale@nariindia.org
National AIDS Research Institute		
Plot # 73, Block G,		
MIDC Bhosari, Pune, 411 026, India		
**Principal Investigator (TRC)**		
V.D. Ramanathan MBBS, PhD	Telephone:	+ 91 44 2836 9650
(London),	Fax:	+ 91 44 2836 9721
Scientist F,	Mobile:	+ 91 94452 59508
Department of Clinical Pathology	E-Mail:	vdrnathan@yahoo.com
Tuberculosis Research Centre		
Chetpet, Chennai, 600 031, India		
**Sponsor Medical Monitor**		
Sonali Kochhar, MD	Telephone:	+ 91 11 2465 2668/9
Medical Director	Fax:	+ 91 11 2464 6464
International AIDS Vaccine Initiative	Mobile:	+ 91 98108 48944
(IAVI)	E-mail:	skochhar@iavi.org
193 1st Floor Jor Bagh		
New Delhi, 110003 India		

Sponsor Medical Expert

Jean-Louis Excler, MD	Telephone(0):	+41 22 369 1391
Senior Medical Director	Mobile:	+41 76 418 4269
International AIDS Vaccine Initiative (IAVI)	E-mail:	jlexcler@iavi.org
11A Route de Duillier		
1270 Trelex		
Switzerland		

Sponsor Medical Director

Patricia Fast MD, PhD	Telephone:	+1 212 847 1068
Executive Director, Medical Affairs	Fax:	+ 1 212 847 1113
International AIDS Vaccine Initiative (IAVI)	Mobile:	+ 1 646 206 6611
110 William Street, 27th Floor	E-mail:	pfast@iavi.org
New York, NY 10038-3901, USA		

Data Coordinating Center

The EMMES Corporation	Telephone:	+ 1 301 251 1161
401 N. Washington Street	Fax:	+ 1 301 251 1355
Suite 700,	E-mail:	iavitrials@emmes.com
Rockville, MD 20850 USA		

IAVI Core Laboratory

Professor Frances Gotch	Telephone:	+ 44 20 8746 8257/8
Department of Immunology	Fax:	+ 44 20 8746 5997
Faculty of Medicine, Imperial College	E-mail:	f.gotch@imperial.ac.uk
Chelsea and Westminster Hospital		
369 Fulham Road		
London SW10 9NH, United Kingdom		

Jill Gilmour, PhD	Telephone:	+ 44 20 8746 5098
Senior Director, Clinical Research	Fax:	+ 44 20 8746 5096
International AIDS Vaccine Initiative, Core Laboratory	Mobile:	+ 44 78 10 202 699
5th Floor St Stephen's Centre	E-mail:	jgilmour@iavi.org
Chelsea and Westminster Hospital		
369 Fulham Road		
London SW10 9NH		

Tony Tarragona, PhD<br>Lab Manager/Administrator<br>IAVI Core Lab<br>Imperial College London<br>Faculty of Medicine<br>St. Stephen's Centre, 5th Floor<br>Chelsea Campus<br>369 Fulham Road<br>London SW10 9NH, United Kingdom	Telephone:<br>Fax:<br>Mobile:<br>E-mail:	+ 44 20 8846 6300<br>+ 44 20 8846 6301<br>+ 44 7940 510090<br>t.tarragona@imperial.ac.uk

Trial Monitoring India

Head – Clinical Trial Operations<br>Reliance Clinical Research Services Pvt. Ltd. (RCRS)<br>Dhirubhai Ambani Knowledge City – Life Science Centre<br>Thane Belapur Road, Rabale<br>Navi Mumbai – 400 701, India	Telephone:<br>Mobile:<br>Fax:<br>E-mail:	+ 91 22 6767 8206 (direct)<br>+91 98 6761 0890<br>+ 91 22 6767 8299<br>jamila.joseph@relclin.com

---

Sponsor's Clinical Program Manager

Marietta Krebs, MHSA, MBA<br>Clinical Program Manager<br>International AIDS Vaccine Initiative (IAVI)<br>50 Beale Street, Suite 1300<br>San Francisco, CA 94105	Telephone:<br>Fax:<br>Mobile:<br>E-mail:	+ 1 415 597 9147<br>+ 1 415 597 9327<br>+ 1 415 425 1122<br>mkrebs@iavi.org

---

2.0 SIGNATURE PAGE

The signatures below constitute the approval of this protocol and the appendices and provide the necessary assurances that this study will be conducted in compliance with the protocol, GCP and the applicable regulatory requirement(s).

Sponsor:

Signed: ______________________________ Date: ______________

Dr. Patricia Fast
Director, Medical Affairs, IAVI

Signed: ______________________________ Date: ______________

Medical Monitor:

Principal Investigator:

Signed: ______________________________ Date: ______________

Name (please print): ______________________________

Name of institution (please print): ______________________________

Principal Investigator:

Signed: ____ Date: _______________

Name (please print): __________________________

Name of institution (please print): _______________

Instructions: The Principal Investigator at the study site will sign and date two copies of the protocol signature page indicating that he/she agrees to conduct the study in accordance with the protocol.

One copy of the original, signed protocol signature page will be returned to IAVI where it will be archived. The other copy of the original signed and dated protocol signature page must be filed in the investigator's site file.

3.0 BACKGROUND INFORMATION

In June 1981, the Center for Disease Control (CDC) in the United States reported the first clinical evidence of a disease that would become known as Acquired Immunodeficiency Syndrome (AIDS). Twenty years later, the AIDS epidemic has spread all over the world. Since the beginning of the epidemic approximately 70 million people have been infected. Globally more than 33.2 million people are living with HIV infection today (2007). In Asia, the estimate was 8.3 million people HIV-infected of whom 2.4 million were women[2]. Over 90% of new HIV infections occur in developing countries, with the majority of infections found in Sub-Saharan Africa and South East Asia. There is an urgent need to explore approaches to control the epidemic, in particular, preventive measures such as health education, treatment of sexually transmitted diseases, preventive vaccines and topical microbicides.

Since the first report of HIV infection among sex workers in Chennai in 1986, it is estimated by the National AIDS Control Organisation (NACO) that between 2 million and 3.1 million people have been infected with HIV in India at the end of 2006, representing a high burden of infection. HIV infection has spread to all the states in India and high prevalence rates have been reported from Maharashtra, Karnataka, Tamil Nadu, Andhra Pradesh, Manipur and Nagaland. A majority of HIV infections are due to heterosexual transmission, followed by intravenous drug use and mother-to-child transmission[3]. HIV epidemic has been spreading from high risk to low risk populations and from urban to rural areas during the last 12-13 years [4 5 6 7].

New infections occur mainly in young people, women, infants of HIV-infected mothers, and economically unprivileged communities. Considerable heterogeneity exists with respect to rate of spread to various sub-populations in various geographic regions in India. This might be attributed to risk factors associated with the spread of HIV and AIDS such as migration, economic instability, drug abuse, sexually transmitted diseases, low levels of literacy, and poverty[8]. Available data suggest that HIV- 1 subtype C is the most predominant circulating subtype in India[9 10 11 12]. The development of a vaccine against the human immunodeficiency virus type 1 (HIV-1) is the main hope for controlling the HIV/AIDS pandemic[13].

The HIV vaccine strategies that are currently being investigated actively in clinical trials include subunit proteins and peptides, recombinant live bacterial and viral vector based vaccines and DNA vaccines. Subunit vaccines, i.e. highly purified recombinant HIV-1 envelope proteins or synthetic peptides, have to date not elicited strong virus-specific CTL nor antibody responses that can neutralize primary isolates of HIV-1, even when used with adjuvants with potent immunostimulants[14 15]. Results of the first efficacy trial of a subunit containing subtype B (www.vaxgen.com) and the Phase II test of concept trial with adenovirus type 5 subtype B (www.merck.com) indicated no overall vaccine efficacy. New vectors such as an attenuated live virus or bacteria carrying genetic material representing a portion of the HIV may prove useful for HIV vaccines[16 17]. Another new approach has been the use of genetically engineered plasmid DNA to direct the synthesis of an immunogen within the host cells[18 19 20].

To date, two HIV vaccine trials have been carried out in India, both testing vaccines that are based on Clade C, the predominant circulating subtype of HIV-1 in India. One studied an AAV-based HIV vaccine; this vaccine has not been proposed for further study in India at this time, as the immunogenicity was not sufficient (Van Lunzen, Clumeck and Mehendale, 2008, AIDS Research and Human Retroviruses, in press). The second candidate vaccine, TBCM4, is proposed for further study. Safety of TBCM4 at two dosage levels was acceptable and immune responses were found in 75% of volunteers after the three administrations at the lower dosage studied, but the magnitude of the responses was modest (Ramanathan et al, manuscript in preparation). Studies by other groups have shown that priming with DNA-

based HIV vaccines can increase the magnitude of responses to poxvirus-vectored HIV vaccines [48,62,63]. Now we propose to study the potential for ADVAX, a DNA-based vaccine representing 5 HIV genes, to augment the responses to TBCM4. ADVAX has been studied in a Phase 1 clinical trial, and the safety of the vaccine was acceptable [68].

3.1. Study Rationale

An ideal HIV vaccine should induce neutralizing antibodies, CD4+ helper T-cells and CD8+ cytotoxic T-cells. Initial vaccine strategies aimed at this goal by applying the prime-boost concept to AIDS vaccines[21]. The basic prime-boost strategy involves priming the immune system to a target antigen delivered by one vaccine and then selectively boosting this immunity by repeat administration of the antigen in the context of a second and distinct vaccine. The key strength of this strategy is that greater levels of immunity are established by heterologous prime-boost than can be attained by a single vaccine administration or homologous boost strategies. This synergistic enhancement of immunity to the target antigen is reflected in an increased number of antigen-specific T-cells, selective enrichment of high avidity T-cells and increased efficacy against pathogenic challenge[22 23]. Synergistic immune responses have been demonstrated in many examples in animals and humans as described below.

Repeated administrations with the same vaccine (homologous boosting) have proven very effective for boosting humoral responses. However, this approach is relatively inefficient at boosting cellular immunity because prior immunity to the vector tends to impair robust antigen presentation and the generation of appropriate inflammatory signals. One approach to circumvent this problem has been the sequential administration of vaccines using different antigen delivery systems or heterologous boosting. Indeed, most of the initial prime-boost vaccines used priming with a viral vector to induce cell-mediated immune responses and boosting with a recombinant envelop protein to induce neutralizing antibodies[24 25]. This concept is now in phase III trial in Thailand[26]. Older approaches used priming with an envelope glycoprotein boosted by subunit peptides[27].

While the induction of broadly neutralizing antibodies remains a highly challenging goal[28], another major challenge for immunologists has been the development of vaccines designed to induce potent and protective cell-mediated responses in animal models[29] and humans[30]. Recent experimental observations suggest approaches to immunization that might finally result in at least a partially effective vaccine against infection with HIV-1. In particular, advances in our understanding of the contribution of vaccine-elicited cellular immunity to protecting memory CD4+ T-cells from virus-mediated destruction and prolonged survival in vaccinated animals provide rational strategies for the development of this vaccine[31 32 33 34].

Vaccines designed to induce cell-mediated immune responses against HIV-1 are unlikely to provide sterilizing immunity but may be associated with reduced viral set points after infection. Recent studies in HIV-infected individuals show that the time to key clinical events in the course of HIV-1 disease progression was significantly extended for those with viral set points 0.5-1.25 log(10) copies/mL lower than the reference group. By quantifying the anticipated clinical benefits associated with a reduction in viral set point, these findings support the use of viral end points in HIV-1 vaccine trials[35]. The goal of an AIDS vaccine regimen designed to induce cellular immune responses should therefore be to reduce the viral set point and preserve memory CD4+ lymphocytes. In non-human primates, vaccine-induced cellular immunity in the absence of any Env-specific antibodies can control viral replication following multiple low-dose challenges with the highly pathogenic SIVmac239 isolate. Vaccine-induced cellular immune responses may, therefore, exert a measure of control over replication of the AIDS virus in the complete absence of neutralizing antibody and give us hope that a vaccine designed to induce cellular immune responses might control viral replication[36 37 38 39].

One particularly promising approach is the prime-boost strategy with DNA[40 41] and vectors or heterologous vectors, which has been shown to generate high levels of T-cell memory and some level of protection in animal models.

Vaccine strategies that involve primary vaccinations with a DNA vaccine followed by boosting with a recombinant poxvirus vector encoding the same immunogen have emerged as favored approaches for generating protective CD8+ T-cell responses against a number of diseases including HIV[42 43 44 45 46 47 48], malaria[49 50 51], tuberculosis[52], and cancer[53 54]. The immunological mechanisms underpinning the seemingly unparallel ability of DNA/poxvirus vaccine regimens to induce enhanced levels of CD8+ T-cell-mediated protection have not been extensively explored. Several explanations have been proposed including the induction of immune responses with enhanced INF-γ secretion phenotypes of Th1 bias, importantly the ability to avoid induction of anti-vector responses by priming with a DNA vaccine and of high avidity CD8+ T-cells. Using challenge conditions to mimic human sexual transmission, a multi-protein DNA/MVA HIV-1 vaccine was indeed capable of protecting rhesus monkeys against systemic infection when challenged repeatedly with a highly heterologous immunodeficiency virus (SHIV). Furthermore, this repetitive challenge approach allowed calculating per-exposure probability of infection, an observed vaccine efficacy of 64% providing novel insight and expectations for positive outcomes of human HIV clinical trials[55].Other promising prime-boost strategies have been developed with DNA[56] and adenovirus[57], Sendai[58], adeno and pox virus vaccines[59], BCG and Vaccinia[60], and fowlpox and MVA[61].

Recently in humans, a phase I trial showed that a prime-boost regimen of DNA and MVA was well-tolerated, induced Elispot INF-g in 11/36 (31%) after DNA alone and in 33/36 (92%) after MVA boost despite using heterologous priming and boosting antigens [DNA: gp160 (A,B,C), gag (A,B), rev & RT (B) MVA: gp150 (E), gag, RT, protease (A)] [62]. Similarly, a regimen with DNA and NYVAC (attenuated replicative vaccinia) showed induction of Elispot INF-g in 6/15 (40%) of the vaccinated subjects after NYVAC alone, very few responders after DNA alone (~10%) and 18/20 (90%) positive after DNA + NYVAC[63]. These data with those generated by Goonetilleke *et al.*[48] suggest that priming is possible with DNA vaccine and that the lack of detectable immune responses after DNA vaccination does not preclude the effective priming of the immune system as demonstrated by the detection of immune responses following boosting, stronger than after either priming or boosting alone. Other similar approaches are developed[64].

The effort to develop an effective preventive vaccine against HIV-1 infection is challenged by the wide genetic diversity of HIV-1 among different isolates. Recent studies suggest priming of T-cell immunity to prevent AIDS in humans is possible, but differences in the immunogenicity of various subtype vaccines and broad cross-subtype protection may still be substantial hurdles[65]. However, using heterologous HIV immunogens derived from different clades for sequential priming and boosting predominantly stimulated T-cell immunity against conserved epitopes, whereas a single vaccine derived from one clade or the mixture of multiple vaccines from different clades primarily raised T-cells against less conservative or non-conservative epitopes[66].

This protocol proposes studying a prime-boost vaccine approach designed mainly to induce cell-mediated immune (CTL) responses. Two vaccine candidates will be used in two different prime-boost regimens: ADVAX (DNA) + TBC-M4 (MVA) and TBC-M4 (MVA) + TBC-M4 (MVA). Both these vaccines have already been tested in humans where no safety concerns were identified and the vaccines were well tolerated and immunogenic. No SAE was found to be related to any of the vaccine products.

This design is hypothesis-driven:

- DNA + MVA is already known to be safe and to induce robust cell-mediated immune responses in non-human primates and humans
- MVA induces both specific CD8+ and CD4+ T-cell responses
- A prime-boost regimen may broaden the immune response to several proteins and epitopes and confer a greater magnitude and longer duration of this response

TBC-M4 (MVA) at 5 x 10^7 pfu has been shown to be safe and immunogenic in Indian volunteers. In a dose-escalation, double-blind, randomized, placebo controlled study (IAVI D001), 32 healthy, HIV-uninfected volunteers received intramuscular injections of TBC-M4 at 0, 1 and 6 months at either $5x10^7$ pfu (n=12) (low dose LD) or $2.5x10^8$ pfu (n=12) (high dose HD) or placebo (n=4 in each group). T-cell responses were assessed on frozen (results presented) or fresh cells by IFN-gamma ELISPOT assay, and anti-vaccinia binding antibody titers (bAb) by vaccinia-specific ELISA assay. HIV testing was made by routine ELISA and DNA PCR. The vaccine appears to be well tolerated after both initial and boost vaccinations. Mostly mild local and systemic reactogenicity was experienced by approximately 53% and 78% of volunteers respectively. Routine laboratory tests remained generally within normal range. No vaccine-related serious adverse events were reported. The proportion of systemic reactogenicity and adverse events were evenly distributed among the different dose groups, whereas higher rate of local reactogenicity was observed in the high dose group. No cardiac event was reported following vaccination with TBC-M4. At baseline, 8/31 (3/15 low dose, 5/16 high dose) of volunteers had detectable bAb against vaccinia. Dose-dependent HIV-specific T-cell responses were detected in 8/12 (67%) and 11/12 (92%) of volunteers after two injections, and in 9/12 (75%) and 12/12 (100%) of the volunteers after the third injection of the LD and HD, respectively. Most of the responses were directed to gag and env epitopes and the magnitude was moderate (39-430 SFC/10^6 PBMC), although persistence of response across multiple timepoints was observed[67].

In a prime-boost regimen, TBC-M4 as a boost may generate a higher number of responders, and a broader immune response of longer duration. The choice of 2 booster or primer injections is made because higher responses are observed after 2 injections. However, it is proposed to analyse responses after one injection as well.

DNA vaccination, or genetic immunization, is an emerging strategy in vaccine development. Briefly, the strategy involves the direct injection of DNA encoding viral antigens into skin or muscle. Local cells then take up the plasmids and express the foreign proteins themselves, essentially manufacturing the vaccine immunogens *in situ*. As it is known that cell-mediated immunity (CMI) is critical for controlling SIV and HIV-1 replication *in vivo*, the generation of CMI is a desirable goal of vaccination. As a consequence of the intracellular synthesis and subsequent protein expression, DNA vaccination appears to allow for antigen to induce CMI. In one study, DNA vaccination resulted in at least partial protection of rhesus macaques from experimental challenge with pathogenic SHIV. DNA vaccines are also being used as vaccines against other diseases e.g. malaria.

ADVAX (DNA) at three dosage levels, i.e. 0.2 mg, 1.0 mg and 4.0 mg has been shown to be generally safe, well tolerated and immunogenic. The study (IAVI C001) was conducted at two sites, The Rockefeller University Hospital, New York and University of Rochester Medical Center, Rochester, New York. In the dose-escalation, double-blind, randomized, placebo-controlled study, 45 healthy, HIV-seronegative volunteers were enrolled in the three dosage groups and randomized in a 4:1 ratio of active vaccine to placebo. The volunteers received three doses of vaccine or placebo intramuscularly at 0, 4 and 12 weeks at the three dosage levels. The vaccine appears to be well tolerated at the three dosage groups. Higher rates of local and systemic reactogenicity were observed in the high dose group. Most laboratory abnormalities were isolated, transient, mild and resolved spontaneously. No vaccine-related serious adverse events were reported. The percentage of volunteers who

had vaccine-induced HIV-1 specific T cell responses detectable by IFN-γ ELISPOT assay in at least one peptide pool on at least one occasion was 25%[68 69].

The clinical safety of ADVAX was excellent at all doses (4 mg, 1 mg and 0.2 mg): there were no severe systemic reactions and an equivalent number of moderate reactions in the high and mid dose groups (17% in each group). Local symptoms were primarily self-limited pain and tenderness; one high dose participant reported severe pain and tenderness and 25% reported moderate tenderness, as compared to 8% with moderate tenderness in the mid dose group. These reactogenicity profiles are considered sufficiently similar that either dose could be selected for further study.

ADVAX induced little or no Elispot response (gamma-interferon secreting T cells) or antibodies at the times tested. In this study, however, the goal is to induce T cell priming that will potentiate a subsequent response to TBCM4, the MVA-based vaccine. Even in the apparent absence of direct responses to DNA-based vaccines, priming has been demonstrated[48]. Because this 'hidden priming' can only be measured in a prime-boost trial, it seems prudent to optimize the chance of detecting it by using the highest dose available for ADVAX.

The 6-month immunization schedule is the usual public health vaccination regimen. It may allow maximizing the immune response at the earliest possible.

The study hypothesis is that the safety and immunogenicity of 2 dose DNA followed by 2 dose MVA strategy is superior to 3 dose MVA vaccine strategy in India where HIV-1 subtype C is most commonly prevalent and both the vaccines are based on subtype C.

4.0 STUDY OBJECTIVES

4.1 Primary Objectives

Primary
To evaluate the safety of ADVAX administered intramuscularly at time 0 and 1 month, followed by TBC-M4 administered intramuscularly at months 3 and 6, compared with TBC-M4 administered intramuscular at 0, 1, and 6 months.

Secondary
To evaluate the immunogenicity of ADVAX administered intramuscularly at time 0 and 1 month, followed by TBC-M4 administered intramuscularly at months 3 and 6, compared with TBC-M4 administered intramuscularly at 0, 1, and 6 months.

Other
To examine the safety and immunogenicity of the construct in the presence of pre-existing immunity against vaccinia, if any participants have such immunity

5.0 STUDY ENDPOINTS AND STUDY DESIGN

5.1 Study Endpoints

5.1.1 Primary endpoints

Safety

- Proportion of volunteers with Grade 3 or 4 local reactogenicity events (injection site pain, tenderness, pruritis, cutaneous reaction, erythema, induration)

- Proportion of volunteers with Grade 3 or 4 systemic reactogenicity events (fever, fatigue, malaise, chills, pain, nausea, vomiting, myalgia)
- Proportion of volunteers with other Grade 3 or 4_adverse events (including laboratory abnormalities)
- Proportion of volunteers with Serious Adverse Events

5.1.2 Secondary endpoints

Safety and tolerability

- Proportion of volunteers with other adverse events.

Immunogenicity

- Proportion of volunteers with HIV-1 specific T-cell responses qualified by ELISPOT assay
- Proportion of volunteers with antibodies to HIV antigens
- Proportion of volunteers with binding antibodies to vaccinia
- Proportion of volunteers showing *in vitro* viral inhibition

5.2 Study Design

Group	N (vaccine/ placebo)	Months			
		0	1	3	6
A	12/4	ADVAX	ADVAX	TBC-M4	TBC-M4
B	12/4	TBCM4	TBC-M4		TBC-M4

The study is a placebo-controlled, randomised, double-blinded, study with respect to allocation of vaccine or placebo, but is not blinded with respect to the group assignment. Thirty-two (32) volunteers will be enrolled, 24 receiving the vaccine and 8 the placebo. It is planned to enroll equal numbers of volunteers at two sites. Both groups will be initiated simultaneously. Safety and tolerability of the vaccine/ placebo will be evaluated by the Data Safety Monitoring Board (DSMB) when half the volunteers have completed their vaccination schedule

5.2.1 Duration of study

Volunteers will be screened up to 42 days before vaccination and followed for 12 months after receiving their last vaccination (18 months total). It is anticipated that it will take 6 months to enroll all volunteers in the study and a further 12 months for follow-up evaluation after the last vaccination. Thus, the total duration of the study would be approximately 24 months (6 month enrollment period + 6 month vaccination period + 12 month follow-up period).

5.2.2 Study population

The study population is composed of healthy male and female volunteers 18 to 50 year old who are not infected with HIV; who are available for the duration of the trial or available to attend scheduled visits if living outside Chennai or Pune; understand the study and can provide written informed consent. The total number of volunteers to be included in the study is 32 (24 vaccine recipients and 8 placebo recipients) to be enrolled at NARI and TRC sites.

5.2.3 Inclusion criteria

1. Healthy males and females as assessed by a medical history, physical examination and laboratory test
2. Age at least 18 years on the day of screening and have not yet reached his/ her 51st birthday on the day of first vaccination
3. Willing to comply with the requirements of the protocol and available for follow-up for the planned duration of the study (screening plus 18 months, see schedule of procedures).
4. In the opinion of the Principal Investigator or designee, has understood the information provided. Written informed consent needs to be given before any study-related procedures are performed.
5. Willing to undergo HIV testing, HIV counseling and receive HIV test results
6. If sexually active female, using an effective method of contraception (combined oral contraceptive pill; injectable contraceptive; intra-uterine device; condoms; anatomical sterility in self or partner) from screening until four months after last vaccination. All female volunteers must be willing to undergo urine pregnancy tests at time points as indicated in the schedule of procedures (Appendix A).
7. If sexually active male, willing to use an effective method of contraception (such as condoms) from enrolment until four months after last vaccination.
8. Willing to forgo donations of blood, sperm, eggs, bone marrow or organs during the study and for those who test HIV positive after vaccination, till the anti-HIV antibody titers become undetectable.

5.2.4 Exclusion criteria

1. Confirmed HIV-1 or HIV-2 infection
2. Any clinically relevant abnormality on history or examination including history of immunodeficiency or autoimmune disease; use of systemic corticosteroids (the use of topical steroids is permitted), immunosuppressive, antiviral, anticancer, anti-tuberculosis, or other medications considered significant by the investigator within the previous 6 months.
3. Any clinically significant acute or chronic medical condition that is considered progressive or in the opinion of the investigator would make the volunteer unsuitable for the study.
4. Any of the following abnormal laboratory parameters listed below:

   - Hematology
     - Hemoglobin <11.0 g/dL
     - Absolute Neutrophil Count (ANC): ≤ 1000/mm^3
     - Absolute Lymphocyte Count (ALC): ≤ 600/mm^3
     - Absolute Eosinophil Count (AEC): ≥ 1500/mm^3
     - Platelets: decreased ≤ 100,000/ mm^3
   - Chemistry
     - Creatinine: > 1. 1 x ULN
     - AST: >1.25 x ULN
     - ALT: >1.25 x ULN
     - Serum albumin <3 g/dL

    - Fasting blood glucose > 110 mg/ dL
    - Glycosylated hemoglobin A1C: > 7%
  - Urinalysis: 3+ confirmed by urine dipstick
    - Protein
    - Blood (not due to menses)
    - Leukocytes
  - Stool examination evidencing helminthiasis infection
  - Cardiac troponin I: > Upper Normal Limit

5. Reported high-risk behaviour for HIV infection, defined as within 6 months before vaccination, the volunteer has:
    - Had unprotected vaginal or anal sex with a known HIV infected person or a casual partner (i.e. no continuing established relationship)
    - Engaged in sex work for money, drugs or shelter
    - Used injection drugs
    - Acquired a sexually transmitted disease (STD) e.g., gonorrhoea, chlamydia, syphilis, *Trichomonas vaginalis, Herpes genitalis.*
    - Had a high-risk partner
6. If female, pregnant or planning a pregnancy within 4 months after last vaccination; or lactating
7. Presence of double stranded DNA antibodies.
8. Receipt of live attenuated vaccine within the previous 60 days (live attenuated flu vaccine within 14 days) or other vaccine within the previous 14 days or planned receipt within 14 days after vaccination with Investigational Product. Prior receipt of smallpox vaccination should be documented, but will not be an exclusion criterion.
9. Receipt of blood transfusion or blood-derived products within the previous 6 months
10. Participation in another clinical trial of an investigational product currently, within the previous 3 months or expected participation during this study
11. Former or ongoing participation in another AIDS vaccine clinical trial
12. History of severe local or systemic reactogenicity events to vaccines or history of severe allergic reactions
13. Confirmed diagnosis of hepatitis B or hepatitis C (HB surface antigen, HCV antibodies) or active syphilis or active tuberculosis.
14. History of severe neurological and/ or psychiatric disorder, or substance abuse.
15. ECG with clinically significant findings or features that would interfere with the assessment of myopericarditis including but not limited to:
    - conduction disturbance (atrio-ventricular or intra-ventricular condition, left or right bundle branch block, AV block of any degree, or QTc prolongation)
    - repolarization (ST segment or T wave) abnormality
    - significant atrial or ventricular arrhythmia
    - frequent atrial or ventricular ectopy (e.g. frequent premature atrial contractions, 2 premature ventricular contractions in a row)
    - ST elevation consistent with ischemia
    - evidence of past or evolving myocardial infarction
16. History of, or known active cardiac disease including but not limited to:
    - previous myocardial infarction (heart attack)
    - angina pectoris
    - congestive heart failure
    - valvular heart disease including mitral valve prolapse
    - cardiomyopathy
    - pericarditis
    - stroke or transient ischemic attack
    - chest pain or shortness of breath with activity (such as walking up stairs)
    - other heart conditions under the care of a doctor

17. Failure in pre-determined psychological assessment test.

5.2.5 Recruitment of study volunteers

Healthy adult male and female volunteers will be recruited through information presented in community organizations, hospitals, colleges and other institutions to the general public. Interested individuals will be provided detailed information about the study and the requirements for participation will be provided. Volunteers will be given volunteer's brochure document. They will have the opportunity to ask any questions they might have and to talk to a counselor. They will be offered to share this information with their spouse and/or family, the decision to do so being left to the choice and discretion of the volunteer. The volunteer will also be encouraged to bring their spouse or family member to attend the counseling session. However, in no case will this procedure be mandatory for enrollment. If they are still interested and willing to participate, they will be invited to participate in screening evaluation procedure. Alternatively, study sites will perform other processes such as one to one sessions with study team members in the pre-screening evaluation phase. Full counseling will be carried out and full informed consent will be obtained.

Study volunteers satisfying all criteria for enrolment after the screening visit will have to pass a test of understanding and may be invited to attend another information seminar if they do not satisfy the test of understanding. They will be offered to enroll in the trial after passing the test of understanding and signing the enrollment consent form. However, repetition of consent process will only be done two times for any particular volunteer.

6.0 STUDY VISITS

6.1 Screening

During the Screening, site personnel will perform the following procedures:

- Provide and/or review the Informed Consent Document and answer any questions about the study prior to obtaining written informed consent.
- Obtain written informed consent prior to conducting any study procedures. In Section 8.1 more information is provided on the Informed Consent process.

If the volunteer agrees to participate, site personnel will:

- Provide a screening questionnaire to the volunteer for completion
- Perform a complete medical history (including concomitant medication)
- Perform a general physical examination including height, weight, vital signs (pulse, respiratory rate, blood pressure and temperature), and examination of skin, respiratory, cardiovascular and abdominal systems, and an assessment of cervical and axillary lymph nodes.
- Ask for history of smallpox vaccination and look for scar of such vaccination
- Conduct pre HIV test counseling
- Perform ECG
- Perform anti-DS DNA test
- Collect blood, urine and stool specimens for all tests as indicated in the Schedule of Procedures (Appendix A).
- Perform a urine pregnancy test for all female volunteers.
- For persons presenting with productive cough of more than 15 days duration, chest X ray and sputum examination for Acid Fast Bacilli will be performed to rule out active tuberculosis.

Screening laboratory test(s) may be repeated at the investigator's discretion to investigate any isolated abnormalities.

If the screening period exceeds 42 days on the date of vaccination, all screening procedures must be repeated. The complete medical history may be replaced by an interim medical history and the Volunteer Information Brochure should be reviewed with the volunteer.

If a volunteer has signed the screening informed consent form but does not meet the eligibility criteria, the records must be kept at the site.

6.2 Vaccination Visit

Prior to the first vaccination, site personnel will:

- Answer any questions about the study
- Review interim medical history (including concomitant medications)
- Review screening safety laboratory data
- Fill in eligibility check list and decide the eligibility of the volunteer to participate in the vaccine trial.

On the day of the first vaccination, site personnel will:

- Review the Enrollment Informed Consent Document with volunteers
- Perform test of understanding with the volunteer
- Obtain signatures on enrollment consent form from the volunteer and from the investigator or assignee
- Perform a directed physical examination including vital signs (pulse, respiratory rate, blood pressure and temperature), examination of vaccination site as well as an assessment of axillary lymph nodes and any further examination indicated by history or observation.
- Conduct pre HIV-test counseling.
- Collect blood and urine specimens for all tests as indicated in the Schedule of Procedures (Appendix A).
- Perform a urine pregnancy test for all female volunteers and obtain results prior to vaccination.
- Conduct baseline assessment of vaccination site and any systemic symptoms.

The volunteer will be assigned an allocation number according to the instructions specified in the Study Operations Manual.

The Investigational Product will be administered as specified in Section 7.6 Administration of Investigational Product.

Site personnel will closely observe volunteers for at least 60 minutes after vaccination for any acute reactogenicity events. At the end of the observation period site personnel will:

- Record vital signs (pulse, respiratory rate, blood pressure and temperature)
- Assess any local and systemic reactogenicity events
- Assess any other adverse events

Volunteers will be kept under observation at the clinical site for 4 hours after injection of the Investigational Product.

If a volunteer has signed the enrollment consent form but does not wish to be enrolled, the records must be kept at the site.

For subsequent vaccination visits, site personnel will perform the same procedures as above with the following exceptions:

- Review the routine safety laboratory parameters (see Section 9.1.5) from the previous visit prior to each vaccination. If a volunteer has an abnormal

laboratory value that is known at the time of vaccination, follow the specified guidelines (see Section 12.1)

- Conduct pre HIV-test counseling if an HIV test is required (Appendix A) or provide post-test counseling if the results of a prior HIV test are being provided to the volunteer.

A volunteer will be considered as ENROLLED once she/he has been randomly allocated to a specific vaccination regimen.

6.3 Post-Vaccination Follow-Up Visits

Volunteers will return to the vaccine trial center once on Day 3 (± 1 day), Day 7 (± 2 days) and Day 14 (± 3 days) post-vaccination.

The site personnel will:

- Review the interim medical history and adverse events (including concomitant medications)
- If symptoms are present, perform a symptom-directed physical examination.
- Assess any local and systemic reactogenicity events
- Collect blood and urine specimens for all tests as indicated in the Schedule of Procedures (Appendix A)
- Perform ECG if required as indicated in the Schedule of Procedures (Appendix A).

In case of adverse event(s), the volunteer will be assessed and followed up by the clinical team. Supplemental visit(s) for further investigation can be planned at the discretion of the clinical and Principal Investigators. Supplemental visit(s) may be recommended if clinically indicated or to clarify observations. All the required supportive care will be provided and referral services will be facilitated.

6.4 Additional Follow-Up Visits

Assessments and procedures will be performed according to the Schedule of Procedures (Appendix A).

6.5 Final Study Visit and Early Termination Visit

Assessments and procedures will be performed according to the Schedule of Procedures (Appendix A).

Site personnel will:

- Review any adverse events and concomitant medications
- Perform a directed physical examination including vital signs (pulse, respiratory rate, blood pressure and temperature), examination of vaccination site and axillary lymph nodes in addition to any further examination indicated by history or observation
- Assess any local and systemic reactogenicity events
- Collect blood and urine specimens for tests.
- Perform a urine pregnancy test for all female volunteers

7.0 INVESTIGATIONAL PRODUCTS

In summary, the two investigational products and their associated placebo are described in the table below:

Description of Investigational Products

Vaccine or Placebo	Dosage level	Total Volume in vial (mL)	Total Volume injected (mL)	Route of Administration
ADVAX	4mg	1.2 mL	1 mL	IM
ADVAX Placebo	NA	1.2 mL	1 mL	IM
TBC-M4	$5x10^6$ pfu	0.75 mL	0.5 mL	IM
TBC-M4 Placebo	NA	0.75 mL	0.5 mL	IM

7.1 Description of ADVAX and placebo

7.1.1 Active vaccine

The ADVAX vaccine consists of one plasmid containing Clade C HIV *env* and *gag* and one plasmid containing Clade C HIV *pol* and *nef/tat* fusion mixed in a 1:1 ratio. The 1mL injection volume provides a 4mg dosage level.

7.1.2 Placebo

The ADVAX placebo is the formulation buffer, which is a sterile phosphate saline buffer, composed of 0.01M sodium phosphate, 150mM NaCl, pH 7.2. ADVAX placebo and ADVAX look similar in appearance.

7.2 Description of TBC-M4 Study Vaccine and Placebo

7.2.1 Active vaccine

The TBC-M4 vaccine manufactured by Therion Biologics Corporation is supplied as a frozen sterile formulation in a 2 mL vial with a butyl stopper and aluminum seal. Lot 1 B which was the low does in the initial clinical study D001 conducted at TRC Chennai will be used for this study. Each vial contains 0.75 mL of vaccine at a concentration of 1×10^7 pfu per mL. The volume of administration is 0.5 mL which will deliver a final dosage of $5x10^6$ pfu. The dose of the vaccine is provided as a virus titer measured as plaque forming units (pfu). The vaccine is formulated in phosphate buffered saline (PBS, sodium chloride, sodium phosphate, potassium chloride and potassium phosphate) with 10% glycerol. TBC-M4 appears as a milky liquid, off-white in color with viral clumps or aggregates.

Transgene (Strasbourg-France) has been identified to continue the titration of TBC-M4 using a method different from the one used by Therion. The stability trial has been re-commenced at Transgene. The lot has been re-issued with new measured potency based on the Transgene assay. The potency values are lower than those formerly provided by Therion but the difference from the nominal potency is due to the different assay technology.Therefore the new titre used is 5×10^6/0.5 m.)

The 5×10^6 pfu dosage level of TBC-M4 is equivalent to the 5×10^7 pfu dosage level used in the previous trial, and is the result of a different assay technology measuring potency.

7.2.2 Placebo

Placebo is provided as a frozen sterile suspension in a 2 mL vial with a butyl rubber stopper and aluminum seal. Each vial contains 0.75 mL of placebo. The volume of administration is

0.5 mL. The placebo is a sterile 0.01M Phosphate Buffered Saline (150mM NaCl) buffer pH 7.0 containing 10% glycerol. The placebo appears as a clear solution.

7.3 Shipment

Authorization to ship the study vaccines and placebos to the site will be provided in writing by the sponsor, upon confirmation that all required critical documents for shipment authorization are completed. The investigational products will be shipped to the sites on dry ice in blinded allocation kits.

7.4 Storage

7.4.1 Storage of ADVAX

The ADVAX e/g + ADVAX p/n-t vaccine and placebo should be stored at -30°C +/- 10°C.

7.4.2 Storage of TBC-M4

The TBC-M4 vaccine and placebo should be stored at - 70°C or below.

7.5 Dispensing and Handling

Complete instructions for handling and administration of investigational products will be supplied in the Study Operations Manual.

7.5.1 Dispensing and Handling of ADVAX

The investigational product will be used as supplied by the manufacturers with no further preparation. Holding the vials in the hand thaws the investigational product. The vials should not be shaken. Study vaccine or placebo is to be administered as soon as possible but not beyond 3 hours post thawing.

7.5.2 Dispensing and Handling of TBC-M4

Each vial containing placebo or vaccine should be thawed at ambient temperature in the pharmacy. Thawed vials should be kept at 2-8°C prior to administration. Vaccine should be utilized within 3 hours of thawing. Immediately prior to drawing into the syringe, the vaccine must be vortexed for 10-20 seconds on high setting prior to drawing into the syringe. While the appearance of clumps will decrease after vortexing, the vaccine may still appear as a suspension of small clumps and will NOT be particle free.

7.6 Administration of Investigational Products

Each Investigational Product will be administered IM according to the Schedule of Procedures (Appendix A). The preferred site of first administration is the deltoid muscle of the non-dominant upper arm (for example, injection in the left arm if volunteer using mainly right arm).

7.7 Accountability and Disposal

All used vials will be accounted for at the end of each vaccination visit, according to the detailed procedures in the Study Operations Manual. The date, allocation number and location of storage of the returned vials will be recorded. During the trial, the product accountability form, the dispensing log and the log of returned vials will be kept and monitored.

At the end of the trial the Study Monitor will check all used and unused vials against the inventory before the vials are returned to the sponsor or destroyed on site, upon written instructions from the sponsor.

8.0 STUDY PROCEDURES

8.1 Informed Consent

The screening and enrolment Informed Consent Documents will be submitted and approved by IAVI and then the Institutional Ethics Committees of TRC and NARI before they can be used at the site.

Volunteer Information
A qualified authorized member of the site personnel will obtain informed consent, after reviewing the volunteer information with the volunteer. In addition to the description of the study procedures for the volunteer, the following study specific elements must be included:

- It is unknown whether or not the Investigational Products will protect against HIV infection or disease
- It may be possible that the vaccinated volunteer will develop antibodies against HIV following vaccination, which may produce a positive result in a routine HIV antibody test, and that provisions have been made to distinguish between response to vaccine and HIV infection during and after the study. In case the volunteer has a positive result in a routine HIV antibody test, he/she will be followed until the result is no longer positive
- A volunteer who is sexually active should use a reliable form of contraception from screening during the vaccination period until 4 months after the last injection.

Understanding of the purpose of the clinical trial and procedures will be tested at the enrollment visit itself before any study procedures are performed. Volunteers will be administered a consent comprehension questionnaire (test of understanding) and those who will be able to answer the appropriate number of questions (as mentioned in the test form) will be able to give their informed consent. For those who fail to qualify, the process will be repeated. If the volunteer fails to qualify on 2 attempts, he/she would be considered ineligible for participation for enrollment.

Informed Consent
All volunteers will give their written consent to participate in the study on the basis of appropriate information and with adequate time to consider this information and ask questions.

The volunteer's consent to participate must be obtained by him/her signing or marking and dating the Consent Form witnessed by a member of the study team. The members of the site personnel who are involved in conducting the informed consent discussions must also sign and date the Consent Form. However, in all cases the investigator or his assignee must be involved in the discussions and should also sign the Consent Form.

The signed and dated consent form must remain at the study site for verification by the monitor. A copy of the signed and dated consent form will be given to the volunteer to take home. Those volunteers who do not wish to take a copy will be required to sign on the form that they declined to take a copy.

Upon request from the volunteer and with their consent, their significant other (s) will be given education and counseling regarding a volunteer’s participation in the trial.

<u>Screening Informed Consent</u>
All volunteers will give their written consent to participate in the screening process.

<u>Enrollment Informed Consent.</u>
All eligible volunteers meeting the criteria to participate in the trial will then give enrollment written consent to participate in the trial.

8.2 Medical History and Physical Examination

At screening, a comprehensive medical history will be collected including details of any previous vaccinations and reaction to vaccinations, history of sexually transmitted diseases, contraceptive practices, and history of epilepsy. At other study visits, an interim medical history will be taken,

A general physical examination includes the following: weight, height, vital signs, and examination of skin, respiratory, cardio-vascular, central nervous and abdominal systems as well as an assessment of cervical and axillary lymph nodes. This will be conducted at screening and termination visits.

At other study visits, a symptom directed physical examination will be performed. A directed physical examination will include vital signs and any further examination indicated by history or observation. Examination of vaccination site will be specifically also done during the reactogenicity assessment period.

8.3 HIV Risk Assessment, HIV Testing and HIV Test Counseling

Study personnel will assess volunteers for past and current risk of HIV infection using the risk assessment questionnaire, screening questionnaire (at the screening visit only).

Additionally site personnel will perform pre HIV test counseling (prior to collecting blood for an HIV test) and post HIV test counseling (when HIV test results are available) as indicated in the Schedule of Procedures (Appendix A). For more information on HIV testing and HIV test counseling, see Section 11.

8.4 Family Planning Counseling

Site personnel will counsel volunteers about the importance of prevention of pregnancies, the use of condoms as well as other effective family planning methods. Condoms and contraceptives will be provided free. Volunteers will be referred to a family planning clinic if a contraceptive prescription is required. The family planning counseling will be performed at time points according to the Schedule of Procedures.

8.5 Blood Collection and Shipment

Between approximately 30 mL and 105 mL of venous blood will be collected per visit usually from the antecubital fossa, according to the Schedule of Procedures (Appendix A). All specimens will be handled according to site-specific and the IAVI Core Laboratory SOPs.

In the event of an abnormal laboratory value, volunteers may be asked to have additional samples collected at the discretion of the Principal Investigator or designee.

8.6 Compensation for Participation

Volunteers will be compensated for the cost of their travel expenses, food, loss of daily wages and any other study-related incidental expenditure (upon documented request) as outlined in the Informed Consent Document. Reimbursement will be provided at the time of each completed scheduled protocol visit. The amount will be decided in consultation with Ethics Committees of NARI and TRC

8.7 Randomization and Blinding

Volunteers will be identified by a volunteer identification number.

Both vaccination groups will be initiated simultaneously. The randomization schedule for the vaccination group, vaccine and placebo will be prepared by the statisticians at the Data Coordinating Center (DCC) prior to the start of the study for both the sites. Volunteers will be assigned a specific allocation number. An unblinding list will be provided to the site by the DCC for emergency use only.

This study is double-blinded. Study staff (investigators and clinical personnel monitoring the safety and laboratory assay result) and volunteers will be blinded with respect to the allocation of investigational product (placebo or vaccine), but not to the group assignment.

Since vaccine and placebo may differ in their physical aspect, a pharmacist will prepare the syringe and deliver it to the person who injects the investigational product. The assessment of safety and reactogenicity will not be performed by the person who injects the investigational product but by the investigators. The placebo to be used in each group will be the formulation buffer of each vaccine.

8.8 Unblinding Procedure

Unblinding of an individual volunteer is indicated only in the event of a medical emergency for which the clinical management and medical treatment of the volunteer would be altered by knowledge of the allocation of investigational product (placebo or vaccine).

The reasons for unblinding should be documented and the DCC should be notified. The unblinding list provided to the site must be returned to the DCC at the end of the trial. The procedures and contact numbers for unblinding are outlined in the Study Operations Manual.

If the study volunteer is unblinded and becomes aware of the allocation of vaccine or placebo assignment, further administration of the investigational product (vaccine or placebo) will be discontinued. The study volunteer will be followed up until the end of the study.

9.0 ASSESSMENT

9.1 Safety Assessment

Data on local and systemic reactogenicity events listed below will be collected by a structured interview, asking specifically about the presence of the events. Data on other adverse events will be collected with an open question.

9.1.1 Local reactogenicity events

The presence of local reactogenicity events will be assessed at the time points specified in the Schedule of Procedures (Appendix A).

Local reactogenicity (injection site pain, tenderness, pruritis, cutaneous reaction, erythema, induration) will be assessed and graded according to Appendix B, Adverse Event Severity Assessment Table.

9.1.2 Systemic reactogenicity events

The presence of systemic reactogenicity will be assessed at the time points specified in the Schedule of Procedures (Appendix A).

Vital signs (pulse, respiratory rate, blood pressure and temperature) will be measured by site personnel prior to vaccination and at least 30 minutes post-vaccination.

Feverishness, chills, headache, nausea, vomiting, malaise and arthralgia will be assessed and graded using the Appendix B, Adverse Event Severity Assessment Table as a guideline.

Other systemic reactogenicity (e.g., chest pain, fatigue, rash, arthralgia, allergic reaction) will be recorded and assessed.

Volunteers will be kept under observation at the clinical site for 4 hours after injection of the Investigational Product.

9.1.3 Other adverse events

Occurrence of other adverse events (including Serious Adverse Events) will be collected following an open question to volunteers on the time points according to the Schedule of Procedures (Appendix A). The adverse events will be graded using the Appendix B, Adverse Event Severity Assessment Table as a guideline.

For more information regarding adverse events refer to Section 10.0, Adverse Events.

9.1.4 Concomitant Medications

During the study, information regarding concomitant medications and reasons for their use will be solicited from the study volunteers at each visit and recorded.

Concomitant receipt of Investigational Products, including other HIV vaccines is prohibited during the study.

If clinically indicated, non-live vaccines (non-HIV) and immunoglobulin may be given up to 14 days before study vaccination(s) or after post-vaccination blood draw (i.e. 2 weeks after study vaccinations).

Live-attenuated vaccines (non-HIV) may be given 60 days before study vaccination(s) or after the post-vaccination blood draw. However, the study vaccination(s) should not be given if there are any continuing symptoms from recently administered non-HIV vaccines. In this situation, the site investigator should consult with IAVI Medical Monitor before administering the next study vaccination.

If the use of a short tapering (<2 weeks) of corticosteroids is required, the study vaccinations may be continued after a 4 week washout period provided that the medical condition requiring this therapy has completely resolved and in the opinion of both the site investigator and IAVI Medical Monitor, the continuation of the study vaccinations will not jeopardize the safety of the volunteer. Volunteers requiring chronic (> 2 weeks) or long term therapy will not receive any further vaccinations but will continue with follow-up visits until the end of the study.

9.1.5 Routine laboratory parameters

Laboratory parameters will routinely include hematology, clinical chemistry, immunological assays, β HCG (for women). Urinalysis and stools examination will be done at the time points indicated in the Schedule of Procedures (Appendix A).

Laboratory parameters

Laboratory Parameter	Test
Hematology	Full blood count (hemoglobin, haematocrit, erythrocytes, leucocytes, platelets)<br>Differential count, absolute neutrophil count, absolute lymphocyte count
Clinical chemistry	Liver function tests (AST, ALT, total and direct bilirubin, albumin),<br>Kidney function test (urea, creatinine)
Immunology	CD4 and CD8 T-cells: absolute counts
Urinalysis	Dipstick test for protein, blood, glucose, ketones, esterase (leukocytes), nitrite.<br>If abnormalities (blood, protein, leucocytes) are found on dipstick test then microscopy will be performed

9.1.6 Specific screening tests

Volunteers will be screened to exclude the following diseases:

- Hepatitis B: positive for hepatitis B surface antigen (HBsAg)
- Hepatitis C: positive for hepatitis C antibodies (HCV antibodies)
- Syphilis: confirmed diagnosis of active syphilis (RPR & TPHA or equivalent)
- Tuberculosis: volunteers presenting with productive cough for more than 15 days duration, a chest X ray and sputum examination for Acid Fast Bacilli will be performed to rule out active tuberculosis.
- Type II diabetes: Fasting glycemia and glycosylated hemoglobin (increasing frequency in South Indian urban areas)
- Helminthic Infection: stool examination for determination of helminthiasis
- Allergic reaction or parasitic infections: Absolute eosinophil count

9.1.7 Cardiac Assessments

Testing for cardiac troponin I will be performed as specified in the Schedule of Procedures (Appendix A). Once a participant has been enrolled, any cardiac troponin result above the institutional upper normal limit should be reported to the sponsor once the results are available and repeated as soon as possible along with a CK-MB (creatine kinase-MB isoenzyme), an erythrocyte sedimentation rate (ESR), and an additional ECG.
A 12-lead ECG will be performed as specified in the Schedule of Procedures (Appendix A).

9.1.8 Double stranded DNA Antibody tests:

Presence and titres of ds DNA antibodies will be determined at screening as indicated in the Schedule of Procedures.

9.1.9 Psychological assessment:

All the prospective vaccine trial participants will be evaluated for their psychological and emotional stability using standardized tools.

9.2 Immunogenicity Assessments

9.2.1 HIV Antibody responses

Blood for antibodies against HIV protein will be taken at time points as indicated in the Schedule of Procedures (Appendix A).

9.2.2 Cellular responses

Blood for immunogenicity assays will be taken at time points indicated in the Schedule of Procedures (Appendix A), and will be evaluated using peptide pools representing all or a portion of the encoded antigen(s). These assays may include ELISPOT for monitoring the number of circulating T-cells that can be stimulated to produce cytokines such as IFN- . Further characterization of phenotype and functional properties of responding T-cells may be performed using flow cytometry and functional assays, including a viral suppression assay, which measures the composite CD8+ T lymphocyte antiviral activity.

It is not anticipated that broadly cross reactive HIV-specific Neutralizing Antibody responses will be induced by the DNA-MVA prime boost regimen outlined in this protocol. If warranted, HIV-specific Neutralizing Antibody responses will be assessed at the time points indicated in the Schedule of Procedures (Appendix A).

Immunological testing for responses to HIV antigens will be performed at the NARI Laboratory and at the TRC Laboratory in accordance with IAVI Core Laboratory standard operating procedures and standard reagents.

At time points indicated in the schedule of procedures (Appendix A), using the procedure provided by the IAVI Core Laboratory, frozen PBMC and sera will be shipped from each site to the IAVI core lab to be tested using ELISPOT assays. These assays are conducted for standardization purposes as mentioned in the Material Transfer Agreement. These samples will be shipped according to an agreed schedule.

9.2.3. Other immunological assessments

Additionally the site laboratories can perform the assays to widen the battery of immunological tests to understand immune response induced by the candidate vaccines using the site specific SOPs with approval of the Institutional Ethics Committee. The data will be made available to IAVI and to the collaborators at the other site.

9.3. PBMC, Serum and plasma storage

Samples for cryopreservation of PBMC, plasma and serum will be taken at time points as indicated in the Schedule of Procedures (Appendix A) for purpose of, standardisation and for future assays related to HIV vaccine research and development. These samples will be archived and only a code will identify the samples.

For the immunogenicity assessment, the laboratory personnel will be trained as necessary by the sponsor and provided with a written procedural manual. The sponsor will also provide specific instructions on reagents.

9.4 Other Assessments

9.4.1 HLA typing

HLA typing may be performed to further characterize the T-cell immune response. This typing would be done by PCR on samples collected at enrollment in a single qualified laboratory jointly designated by the Tuberculosis Research Centre, Chennai, and the National AIDS Research Institute, Pune (mentioned in the Study Operations Manual).

9.4.2 HIV test

Samples will be tested at the time points indicated in the Schedule of Procedures (Appendix A).

9.4.3 Antibody response to the MVA vector

Since pre-existing immunity to vaccinia as well as antibodies induced by MVA itself may impair subsequent immune responses to the proteins of interest expressed by the vector, history and scar of smallpox vaccination will be recorded. Antibodies to the vector will be assessed at time points specified in Schedule of Procedures (Appendix A). Samples for anti-vaccinia neutralizing antibodies will be sent to V-Bio, Inc. St. Louis University, MO, USA for testing [69]. Additional assays for assessment of binding antibodies to MVA may be considered.

9.4.4. Viral inhibition assays

Viral inhibition assays will be carried out at IAVI Core Laboratory at 4 time points indicated in the Schedule of Procedures. The site may carry out these assays at other time points. The methodology and data will be shared with IAVI and the collaborators at the other site.

9.4.5 Pregnancy test

A pregnancy test will be performed by measurement of Human Chorionic Gonadotrophin (β HCG) on urine samples collected from all female volunteers at time points as indicated in the Schedule of Procedures. The results of the pregnancy test must be negative prior to vaccination.

9.4.6 Concomitant medication

Volunteers will be asked about concomitant medication. Each medication taken during the course of the study and the reason for its use will be collected.

10.0 ADVERSE EVENTS

10.1 Definitions

An adverse event (AE) is any untoward medical occurrence during the course of the study in a volunteer administered investigational product and which does not necessarily have a causal relationship with the investigational product. An AE can therefore be any unfavorable or unintended sign (including an abnormal laboratory finding), symptom or disease temporally associated with the use of the investigational product whether or not related to the investigational product.

10.2 Assessment of Severity of Adverse Events

Assessment of severity of all AEs is ultimately the responsibility of the investigator.

The following general criteria should be used in assessing adverse events as Grade 1-4:

- Grade 1: symptoms causing no or minimal interference with usual social & functional activities
- Grade 2: symptoms causing greater than minimal interference with usual social & functional activities
- Grade 3: symptoms causing inability to perform usual social & functional activities
- Grade 4: symptoms causing inability to perform basic self-care functions OR medical or operative intervention indicated to prevent permanent impairment, persistent disability or death

Guidelines for assessing the severity of specific adverse events and laboratory abnormalities are listed in Appendix B, Adverse Event Severity Assessment Table.

10.3 Relationship to Investigational Product

The relationship of an (S)AE is assessed and determined by the Investigator. All medically indicated and available diagnostic methods (e.g. laboratory tests, blood smear, culture, X-ray

etc.) should be used to assess the nature and cause of the AE/SAE. Best clinical and scientific judgment should be used to assess relationship of AE/SAEs to the Investigational Product and/or other cause.

The following should be considered for the assessment of relationship of adverse events to the investigational Product:

- Presence/absence of a clear temporal (time) sequence between administration of the Investigational Product and the onset of AE/SAE
- Presence/absence of another cause that could more likely explain the AE/SAE (concurrent disease, concomitant medication, environmental or toxic factors etc.)
- Whether or not the AE/SAE follows a known response pattern associated with the investigational Product

The relationship assessment should be reported as one of the following:

Not Related: clearly explained by another cause (concurrent disease, concomitant medication, environmental or toxic factors etc.).

Unlikely: more likely explained by another cause (concurrent disease, concomitant medication, environmental or toxic factors etc.).

Possibly: equally likely explained by another cause but the possibility of the Investigational Product relationship cannot be ruled out (e.g. reasonably well temporally related and/or follows a known Investigational Product response pattern but equally well explained by another cause).

Probably: more likely explained by the investigational Product (e.g. reasonably well temporally related and/or follows a known Investigational Product response pattern and less likely explained by another cause).

Definitely: clearly related and most likely explained by the Investigational Product

For the purpose of expedited safety reporting, all possibly, probably or definitely related SAEs are considered Investigational Product related SAEs.

10.4 Serious Adverse Events

An adverse event is reported as a "Serious Adverse Event" if it meets any the following criteria (as per ICH GCP Guidelines):

- Results in death
- Is life threatening
- Results in persistent or significant disability/incapacity.
- Requires in-patient hospitalization or prolongs existing hospitalization.
- Is a congenital anomaly/birth defect or spontaneous abortion.
- Any other important medical condition that requires medical or surgical intervention to prevent permanent impairment of a body function or structure.

Serious Adverse Events (SAEs) should be reported to IAVI within 24 hours of the site becoming aware of the event. All SAEs should be reported using the designated SAE Report Form. The SAE Report should be sent according to SAE Reporting Guidelines (see Study Operations Manual).

To discuss Investigational Product related SAEs or any urgent medical questions related to the SAE, the site investigator should contact one of the IAVI medical monitors directly (see the Contact List).

The IAVI SAE Report Form should be completed with all the available information at the time of reporting. The minimum data required in reporting an SAE are the volunteer identification number, date of birth, gender, event description (in as much detail as is known at the time), onset date of event (if available), reason event is classified as Serious, reporting source (name of Principal Investigator or designee), relationship assessment to the investigational product by the investigator.

The Principal Investigator or designee is required to write a detailed written report with follow up until resolution or until it is judged by the Principal Investigator or designee to have stabilized.

The Principal Investigator or designee must notify the sponsor, local IRB/IEC and local regulatory authorities of all SAEs within the timelines. The sponsor will notify the Data Safety Monitoring Board and the regulatory authorities outside India if applicable and the other study site in India where the same Investigational Product is being tested.

More details on SAE definitions and reporting requirements are provided in the SAE Reporting Guidelines (see Study Operations Manual).

10.5 Clinical Management of Adverse Events

Adverse events (AEs) will be managed by the clinical study team who will assess and treat the volunteer as appropriate, including referral. If any treatment/medical care is required as a result of the harm caused by the Investigational Product or study procedures, this care will be provided free of charge.

If a volunteer has an adverse event and/or abnormal laboratory value that is known at the time of study vaccination(s), the specifications of Section 11.1 will be followed.

Volunteers will be followed until the adverse event resolves or stabilizes or up to the end of the study, whichever comes last. If at the end of the study, an adverse event (including clinically significant lab abnormality) which is considered possibly, probably or definitely related to the Investigational Product is unresolved, follow-up will continue until resolution if possible and the volunteer will be referred.

10.6 Pregnancy

Although not considered an adverse event, if a female volunteer becomes pregnant during the study, it is the responsibility of the Principal Investigator or designee to report the pregnancy promptly to IAVI using the designated case report forms. However, serious complications of pregnancy that meet SAE criteria specified in the Section 10.4 of this Protocol (e.g., eclampsia, spontaneous abortion etc.) should be reported as SAEs. For follow up on a pregnancy refer to Section 12.2.

If a female volunteer becomes pregnant during the study, vaccinations will be discontinued and the volunteer followed until the end of pregnancy or study completion, whichever occurs last. Approximately 2–4 weeks after delivery, the baby will be examined by a physician to assess its health status and the results will be reported to IAVI.

10.7 Intercurrent HIV Infection

HIV infection cannot be caused by the Investigational Product. If a volunteer is found to be HIV-infected through exposure in the community, study vaccinations must be discontinued and the volunteer followed according to procedures described in Section 12.2.

Intercurrent HIV infection in study volunteers, although not considered an SAE must be reported promptly to IAVI using the designated case report forms. However, serious medical conditions associated with the HIV infection that meet SAE criteria specified in the Section 10.4 of this Protocol (e.g. sepsis, PCP pneumonia etc.) should be reported as SAEs using SAE Report Form.

11.0 MANAGEMENT OF HIV ISSUES DURING AND FOLLOWING THE TRIAL

11.1 HIV Risk Assessment, HIV Testing and HIV Test Counseling

HIV testing
Only volunteers who are not HIV-infected at screening will be enrolled into the study.

All HIV screening tests and routine post-vaccination tests will be done at the clinical site laboratory. A predetermined algorithm will be followed to distinguish between an immune response to the vaccine from an HIV infection acquired through exposure in the community.

To prevent unblinding of the volunteer and site personnel, results will be reported to the site as “HIV-infected” or “HIV-uninfected.

- If a volunteer is found to be HIV-infected, a newly drawn blood specimen will be collected for confirmation according to the diagnosis algorithm.
- Volunteers who test positive for vaccine-induced HIV antibodies (HIV-uninfected) will be followed up until the test becomes negative.

Should a volunteer require an HIV test outside the study for personal reasons, it is recommended that the volunteer contact the study staff first. The HIV test can then be performed either at the clinical site laboratory or in an independent laboratory, as appropriate. Written evidence of HIV status (infected or not infected) will be provided upon request.

HIV test counseling
Pre and post HIV test counseling will be provided:

- The pre HIV test counseling process will include information on HIV, safe sex practices and risk reduction. The objective of counseling is to ensure that volunteers have sufficient knowledge about HIV infection, to understand the purpose of the test, the implications of a positive or negative result and the standard of care available locally for HIV infection. Volunteers will be told that it is possible to have a false positive HIV Antibody Test result due to a response to the Investigational Product. Risk reduction counseling will be provided during the study to avoid high-risk behavior for HIV infection.

- Post HIV test counseling will be offered once HIV test results are available or at the next visit.

11.2 HIV infection

Volunteers who are found HIV-infected at screening and volunteers who acquire HIV infection during the study will be managed in the following way:

11.2.1 Counselling

The volunteer will be counselled by the study counsellors. The counseling process will assist the volunteer with the following issues:

- Psychological and social implications of HIV infection
- Whom to inform and what to say
- Implications for sexual partners
- Implications for child-bearing
- Avoidance of transmission to others in future

11.2.2 Referral for support and care

Volunteers will be counseled and referred for support, care and treatment. For the volunteers who acquire HIV infection during the study, care and treatment will be provided according to the National AIDS Control Organization guidelines and IAVI Standard of Care Policy. Any HIV-infected volunteer will be informed of these policies prior to referral. Management of trial product related events will be the responsibility of the trial sponsors. The volunteers will be provided health insurance for the duration of the trial to cover the costs of trial unrelated events. Arbitration Boards will be established at both trial sites to take quick decisions on grievances if any. To ensure the trial participants' safety, the trial site investigators will make arrangements to provide emergency medical care even before the final decision of the Arbitration Board.

The long-term follow-up protocol for all the IAVI sponsored HIV vaccine trial participants is being finalized. This information will be provided to the trial participants during the study enrolment consent procedure. The trial participants will be given an option to consider participation in the long-term study protocol. However, those who are not willing to participate in the long-term follow-up study would still be considered eligible to participate in the current trial.

11.3 Social Discrimination as a Result of an Antibody Response to Investigational Product

The aim of the following precautions is to minimize the possibility of social discrimination in volunteers (if any) who develop vaccine-induced HIV antibodies and therefore test positive on a routine ELISA HIV antibody test.

- Volunteers will be told during the informed consent process that testing positive on a routine HIV antibody test is a potential consequence of participation in this study and that they must be aware of the potential harm a positive test may cause. Each volunteer will receive pre and post HIV test counseling where these issues will be discussed.

- Appropriate diagnostic HIV testing as outlined above and certification for volunteers as required, will be provided both during and after the study.

12.0 DISCONTINUATION OF VACCINATIONS AND/OR WITHDRAWAL FROM THE STUDY

12.1 Discontinuation of Vaccinations

The information regarding any volunteer discontinuing from further vaccinations or being considered for discontinuation of vaccinations will be discussed with the sponsor. Volunteers will be discontinued from further vaccination for any of the following reasons:

1. Pregnancy
2. Intercurrent HIV Infection

3. Use of systemic corticosteroids, immunosuppressive, antiviral, anticancer, or other medications.
4. A disease or condition or an adverse event that may develop, regardless of relationship to the Investigational Product, if the Principal Investigator or designee is of the opinion that further study vaccinations will jeopardize the safety of the volunteer.
5. Any of the following abnormal laboratory parameters which are known at the time of vaccination:

    - Hematology
        - Hemoglobin <10.0 g/dL
        - Absolute Neutrophil Count (ANC): ≤ 1000/mm^3
        - Absolute Lymphocyte Count (ALC): ≤ 600/mm^3
        - Absolute Eosinophil Count (AEC): ≥ 1500/mm^3
        - Platelets: decreased ≤ 100,000/ mm^3
    - Chemistry
        - Creatinine: > 1. 1 x ULN
        - AST: >1.25 x ULN
        - ALT: >1.25 x ULN
        - Serum albumin<3 g/dL
    - Urinalysis: 3+ confirmed by urine dipstick
        - Protein
        - Blood (not due to menses)
        - Leukocytes
6. Receipt of live attenuated vaccine within the previous 60 days (live attenuated flu vaccine within 14 days) or planned receipt within 60 days after vaccination with Investigational Product or receipt of other vaccine within the previous 14 days or planned receipt within 14 days after vaccination with Investigational Product.
7. A severe local reactogenicity involving the major part of the injected arm circumference.
8. Anaphylaxis; bronchospasm; laryngeal oedema; convulsions or encephalopathy following study vaccinations.
9. Life threatening adverse event following study vaccinations unless not related to the Investigational Product and fully resolved.
10. Any immediate hypersensitivity reaction judged to be definitely related to the Investigational Product.
11. Volunteer request to discontinue further vaccination.
12. Participating in another clinical study of an Investigational Product

12.2 Follow-up after discontinuation of vaccinations

Volunteers who discontinue vaccinations for one or more of the reasons in Section 12.1 will remain in the study and all procedures, except vaccination, will be performed according to the Schedule of Procedures (Appendix A) after consultation between the Principal Investigator and the IAVI India Medical Director.

Any adverse event resulting in the discontinuation of a volunteer's vaccinations will be followed until resolution or until the adverse event is judged by the Principal Investigator or designee to have stabilized, when possible.

The pregnant volunteer will be followed to the completion/termination of the pregnancy. If the pregnancy continues to term, approximately 2-4 weeks after delivery, the health of the infant will be examined and reported to the sponsor.

Follow-up of HIV-infected individuals who have received investigational product will be determined by the Trial Working Group and the Medical Director of IAVI India.

The date and reason for discontinuation of vaccination should be recorded.

12.3 Withdrawal from the Study (Early Termination)

Volunteers may be withdrawn from the study permanently for the following reasons:

1. Volunteers who wish to withdraw from the study at any time for any reason.
2. The Principal Investigator or designee has a reason to believe that the volunteer is not complying with the protocol or the volunteer's health might be endangered by continued participation at any point of time.
3. If the sponsor decides to terminate or suspend the study.

12.4. Follow-up Withdrawal from the Study (Early Termination)

If the volunteer withdraws from the study, all termination visit procedures will be performed according to the Schedule of Procedures (Appendix A) where possible. Every effort will be made to determine and document the reason for withdrawal from the study.

13.0 DATA HANDLING

13.1 Data Collection and Record Keeping at the Study Site

Data Collection: All study data will be collected by the clinical study staff using designated source documents and entered onto the appropriate electronic Case Report Forms (eCRFs). Paper forms derived from the eCRFs will be provided by IAVI which can be used as source documents. A file will be held for each volunteer at the clinic(s) containing all the source documents. Source documentation will be available for review to ensure that the collected data are consistent with the CRFs.

All CRFs and laboratory reports will be reviewed by the clinical team, who will ensure that they are accurate and complete.

Source documents and other supporting documents will be kept in a secure location and remain separate from volunteer identification information (name, address etc.) to ensure confidentiality.

Standard GCP practices will be followed to ensure accurate, reliable and consistent data collection.

Source documentation includes but is not limited to:

- Signed Informed Consent Documents
- Dates of visits including dates of vaccinations
- Documentation of any existing conditions or past conditions relevant to eligibility
- Reported laboratory results
- All adverse events
- Concomitant medications
- Local and systemic reactogenicity

13.2 Data Collection and Transfer at the IAVI Core Laboratory

Data generated at the IAVI Core laboratory will be transferred directly to the Data Coordinating Centre.

13.3 Data Entry at the Study Site

The data collected at the site will be either:

- Entered onto the CRFs by the site personnel. To provide for real time assessment of safety, data should be entered as soon as they are available (e.g. within one week of a visit).
- If immunogenicity tests are performed locally, the Immunogenicity assay results will be transferred within 2 weeks of the assay being performed.

13.4 Data Analysis

The data analysis plan will be developed and agreed upon by the sponsor and the Principal Investigator prior to unblinding of the study. The statistician at the Data Coordinating Centre in collaboration with the Principal Investigator and the Sponsor will create tables according to this data analysis plan. Standard Operating Procedure [SOP] document will be developed jointly by IAVI, NARI, TRC and ICMR to outline blinding, coding, data analysis, data sharing, interim analysis, report preparations, abstract and manuscript development and data dissemination procedures.

The DCC will conduct the data analysis and will provide interim and final study reports for the sponsor, Principal Investigator, the DSMB and the regulatory authorities as appropriate. The data will not be released without consultation between the site investigators, ICMR, ADARC and IAVI.

14.0 STATISTICAL CONSIDERATIONS

14.1 Sample Size

The study investigates two groups of 16 volunteers randomized in a 3:1 ratio of active vaccine to placebo.

A total of up to 32 volunteers will be enrolled in the study; 24 volunteers will be given active vaccines and 8 volunteers will be given placebo only. The small sample size is appropriate for an exploratory study of a novel product while safety, tolerability and immunogenicity of the vaccine are investigated.

14.2 Statistical Power and Analysis

Vaccine Safety and Tolerability

The proportions of volunteers with Grade 3 or 4 local reactogenicity events, Grade 3 or 4 systemic reactogenicity events, serious adverse events (SAE) or Grade 3 or 4 adverse events will be used to measure vaccine safety. The proportions of volunteers with moderate local and systemic reactogenicity events and other adverse events will also be assessed. Adverse events that may be temporarily incapacitating (for example, loss or cancellation of work or social activities), which could make a vaccine impractical for large scale use if they occur in more than a small proportion of cases, will also be assessed.

All adverse events will be reported, grouped according to their severity assessment, their relationship to vaccine (as judged by the investigator and reviewed by the sponsor and the DSMB) and whether they are classed as SAE.

Power Considerations

If none of the volunteers receiving an active vaccine experiences an event, the 2-sided 95% upper confidence bound for the rate of these adverse events in the population is 0.17 (n=24)

and 0.31 (n=12), by the Clopper Pearson method. Similarly, if none of the 8 volunteers receiving placebo experiences an event, the 2-sided 95% upper confidence bound is 0.42.

There is limited power for comparison of event rates between treatment arms. For example, with 80% power, the minimum detectable differences in event rates between the placebo arm (n_1 = 8) and the vaccine arms (n_2 = 12 & 24) are 59% and 45%, respectively, assuming a one-percent incidence rate in the placebo arm (one-sided Fisher's exact test with significance level of 0.05).

Planned Statistical Analyses

Analysis of events rates will be conducted using one-sided Fisher's exact test in comparing 2 active vaccine groups against placebo and two-sided Fisher's exact test in contrasting two vaccine groups. A significance level of 0.05 will be used and no adjustment will be made to preserve the overall type I error due to the exploratory nature of this Phase I study.

Vaccine Immunogenicity

Cellular immune responses will be analyzed using binomial methods to examine for the presence or absence of HIV-specific T-cell responses quantified by ELISPOT. Presence or absence of binding and neutralizing antibodies will also be analyzed. Assays will be performed in a similar fashion in all volunteers. Because of the small sample sizes and multiple epitopes, the results will be primarily descriptive.

Power Considerations

The outcome of interest will be the proportion of volunteers with positive responses after the 3rd or 4th vaccinations (boost vaccinations). Given the small group sizes, the response rate in a single group will have to be about 50% or more to be statistically significantly different to the response rate in the placebo group (based on a response rate of 1% in the placebo group – see below).

Contrast between vaccine and placebo groups: The study has at least 80% power (one-sided Fisher's exact test, with ∀ □=0.05) to detect a difference of 59%, 61% and 62% or more in the rate of positive responses between an active group (n_1=12) and the placebo group (n_2=8), assuming the rate of positive responses in the placebo group to be 1%, 5% and 10%, respectively.

Contrast between two active vaccine groups: The minimum detectable difference between the two vaccine groups is 52%, with α=0.05 and 80% power (two-sided Fisher's exact test), based on an assumption of 1% positive response rate in one of the two groups. If the response rate in one group was 10%-and 20%, the minimum detectable difference would be 59% and 60%, respectively (i.e., the corresponding response rates in the other group would have to be 69% and 80%).

Planned Statistical Analyses

Analysis of volunteer positive rates will be conducted using one-sided Fisher's exact test in comparing 2 active vaccine groups against placebo and two-sided Fisher's exact test in contrasting two vaccine groups. A significance level of 0.05 will be used and no adjustment will be made to preserve the overall type I error or for multiple testing due to the exploratory nature of this Phase I study.

15.0 QUALITY CONTROL AND QUALITY ASSURANCE

To ensure the quality and reliability of the data gathered and the ethical conduct of this study, a Study Operations Manual has been developed for all study procedures.

Regular monitoring will be performed according to ICH-GCP. The monitor will ensure that the study is conducted, recorded and reported in compliance with the protocol, ICH/GCP and applicable regulatory requirements. An independent audit of the study may be performed, if appropriate.

The Principal Investigators, by signing the protocol, agree to facilitate study related monitoring, audits, IRB/IEC review and regulatory inspection(s) and direct access to source documents. Such information will be treated as strictly confidential and will under no circumstances be made available publicly.

16.0 DATA AND BIOLOGICAL MATERIAL

All data and all biological material collected through the clinical trial shall be the joint property of the investigators and IAVI.

The raw computerized data generated in this study will be held by the DCC on behalf of IAVI and the investigators. The clinical sites will also hold the frozen data files and tables generated for the purposes of analysis. Investigators will have access to the clinical trial database with appropriate blinding. IAVI and DCC will make site-specific data available to the site investigators on request as outlined in the data sharing SOP.

17.0 ADMINISTRATIVE STRUCTURE

The Principal Investigators will be responsible for all aspects of the study at the trial sites.

17.1 Data Safety Monitoring Board (DSMB)

The DSMB will oversee the progress of the study. The DSMB will consist of independent and highly respected experts including clinician, scientists [vaccine expert], statistician and ethicist who are not involved in the study. Investigators responsible for the clinical care of volunteers or representative of the sponsor will not be the members of the DSMB.

However, the DSMB may invite the Principal Investigator or designee and a sponsor representative to an open session of the meeting to provide information on study conduct, present data or to respond to questions.

The review of study data by the DSMB will take place when half the volunteers have completed their vaccination visits or may be specifically requested (see Section 17.1.2 Indications for Discontinuation of Vaccinations in all Volunteers).

17.1.1 Content of Interim Review

The DSMB will be asked to review the following data:

- All Grade 3 or 4 clinical adverse events/ reactogenicity judged by the Principal Investigator or designee to be possibly, probably or definitely related to the Investigational Product OR

- All Grade 3 or 4 laboratory adverse events confirmed on retest and judged by the Principal Investigator or designee to be possibly, probably, or definitely related to Investigational Product.

- All Serious Adverse Events, independent of relationship to the Investigational Product.

17.1.2 Indications for Discontinuation of Vaccinations in all Volunteers

If 3 or more of the volunteers participating in this study develop an SAE judged definitely, probably or possibly related to the Investigational Product, the Principal Investigator or designee and the sponsor will request a review by the DSMB. The study will be suspended pending a review of all safety data by the DSMB. The study may be unblinded at the discretion of the DSMB.

Following this review, the DSMB will make a recommendation to the Sponsor and the Principal Investigator regarding the continuation of the study.

17.2 Study Supervision

The DSMB and the IAVI Director of Medical Affairs will be provided progress report(s) of this study. Close cooperation between members of the DSMB will be necessary to track study progress, respond to queries about proper study implementation and management, address issues in a timely manner, assure consistent documentation and effective information sharing. Rates of accrual, retention and other parameters relevant to the sites' performance will be monitored regularly and closely by the study team as well as the DSMB.

18.0 INDEMNITY

Trial indemnity and product liability will be covered by IAVI, the trial sponsor. For research-related injuries and/or medical problems determined as resulting from receiving the investigational product, necessary emergency care and treatment and proper follow-up care will be made available to the study volunteer free of charge. Medical Insurance will also be provided free of charge to all enrolled volunteers for the duration of the trial.

19.0 PUBLICATION AND COMMUNICATION

A primary manuscript or abstract for an International Scientific Conference describing safety and immune responses in this trial will be prepared promptly after the (preliminary and final) data analysis is available, based on the data compiled by the IAVI statistical center. Authors will be representatives of the trial site, the statistical center, the laboratories and IAVI, subject to the generally accepted criteria of contributions to the design, work, analysis and writing of the study. Manuscripts and abstracts will be reviewed by representatives of each participating group (TRC, NARI, IAVI, ADARC and Core Laboratory) as specified in the Clinical Trial Agreement.

20.0 ETHICAL CONSIDERATIONS

Each Principal Investigator will ensure that the study is conducted in compliance with the protocol, Standard Operating Procedures in accordance with guidelines laid down by the International Conference on Harmonisation for Good Clinical Practice in clinical studies, the ethical principles that have their origins in the Declaration of Helsinki and applicable regulatory requirements.

The study will be reviewed and approved by the Indian national authorities. The trial will not be initiated before the protocol and Informed Consent Document have been reviewed and received approval/ favorable opinion from the appropriate ethical and regulatory bodies.

APPENDIX A - SCHEDULE OF PROCEDURES

**Visits for Group:**	**A and B**									**B**	**A**				**A and B**						
Study Month (M)	Screening	**M0**				**M1**				M3	**M3**				**M6**				M9	M12	M18 /ET
Study Day (D)		**D0**	**M0 +D3**	**M0 +D7**	**M0+ D14**	**D28**	**M1 + D3**	**M1+ D7**	**M1+ D14**	D91	**D91**	**M3+ D3**	**M3++ D7**	**M3+D 14**	**D182**	**M6+D3**	**M6+ D7**	**M6+D 14**	D273	D364	D546
Visit Windows (days)	- 42		± 1	± 2	± 3	± 3	± 1	± 2	± 3	± 3	± 3	±1	± 2	± 3	± 7	± 1	± 2	± 3	± 7	± 7	± 7
Screening/Enrollment Informed Consent	X	X																			
**Vaccine/Placebo**		X				X					X				X						
Screening Questionnaire	X																				
Test of understanding/ comprehension		X																			
Medical history (including concomitant medication)	X	X	X	X	X	X	X	X	X	X	X	X	X	X	X	X	X	X	X	X	X
General Physical Exam	X	X				X															X
Directed Physical Exam			X	X	X		X	X	X	X	X	X	X	X	X	X	X	X	X	X	
Pregnancy test (women)	X	X				X				X	X				X				X		X
Serum HIV ELISA<br>Pre-/Post test counseling, safe sex, family planning and risk reduction counseling	X	X				X				X	X				X				X	X	X
dDNA antibodies	X																				
Adverse Events		X	X	X	X	X	X	X	X	X	X	X	X	X	X	X	X	X	X		
Serious Adverse Events		X	X	X	X	X	X	X	X	X	X	X	X	X	X	X	X	X	X	X	X
Vital Signs (pre-and post vaccination)		X				X					X				X						
Local and systemic reactogenicity assessment		X	X	X	X	X	X	X	X		X	X	X	X	X	X	X	X			
Tuberculosis (chest x-ray and sputum), Hepatitis B (HBsAg), Hep C (Ab), Syphilis, type II diabetes (fasting hyperglycemia and glycosylated hemoglobin),	X																				
Stool examination for helminthiasis	X																				
Collect samples for possible HLA Typing		X																			
Cardiac Troponin I	X															X					
ECG	X															X					
Hematology - Clinical Chemistry	X	X			X	X			X	X	X			X	X			X	X	X	X
Immunology (CD4 & CD8)	X	X			X	X			X	X	X			X	X			X	X	X	X
Urinalysis	X	X			X	X			X	X	X			X	X			X	X	X	X
Cellular immunogenicity assays		CX		X	X	X		X	C	X	X		X	X	X		X	C	X	C	[X]
Neut. antibodies		C*				X					X				X						[X]
anti-HIV and anti-vaccinia-specific antibodies		C**			X	X			C**	X	X			X	X			C**	X	C**	[X]

Serum, plasma, PBMC storage		X		X	X	X		X	X	X	X		X	X	X		X	X	X	X	[X]

[X] = Optional; C =Time points when IAVI core laboratory would receive samples

CX- samples will be equally shared between the CL and sites

C - samples will be shipped to the Core Lab London for ELISPOT analysis, viral inhibition assay. ELISPOTS will be processed on site for all other timepoints.

C** - If warranted, samples will be shipped to the Core Lab for anti-HIV and anti-vaccinia-specific antibodies.

C* - samples will be shipped to the Core lab for testing at this timepoint, all other neut Abs testing will be done on sites

Calculate each visit date by using number of study days from D0 onwards with the exception of the post-vaccination visits where the number of days are calculated from the most recent vaccination visit date.

APPENDIX B - ADVERSE EVENT SEVERITY ASSESSMENT TABLE

ADAPTED FROM DIVISION OF AIDS TABLE FOR GRADING THE SEVERITY OF ADULT ADVERSE EVENTS
VERSION 1.0, DECEMBER, 2004

CLINICAL				
**PARAMETER**	**GRADE 1**	**GRADE 2**	**GRADE 3**	**GRADE 4**
**ESTIMATING SEVERITY GRADE**				
Clinical adverse event NOT identified elsewhere in this DAIDS AE grading table	Symptoms causing no or minimal interference with usual social & functional activities	Symptoms causing greater than minimal interference with usual social & functional activities	Symptoms causing inability to perform usual social & functional activities	Symptoms causing inability to perform basic self-care functions OR Medical or operative intervention indicated to prevent permanent impairment, persistent disability, or death
**SYSTEMIC**				
Acute systemic allergic reaction	Localized urticaria (wheals) with no medical intervention indicated	Localized urticaria with medical intervention indicated OR Mild angioedema with no medical intervention indicated	Generalized urticaria OR Angioedema with medical intervention indicated OR Symptomatic mild bronchospasm	Acute anaphylaxis OR Life-threatening bronchospasm OR laryngeal edema
Chills	Symptoms causing no or minimal interference with usual social & functional activities	Symptoms causing greater than minimal interference with usual social & functional activities	Symptoms causing inability to perform usual social & functional activities	NA
Fatigue<br>Malaise	Symptoms causing no or minimal interference with usual social & functional activities	Symptoms causing greater than minimal interference with usual social & functional activities	Symptoms causing inability to perform usual social & functional activities	Incapacitating fatigue/ malaise symptoms causing inability to perform basic self-care functions
Fever (nonaxillary)	37.7 – 38.6°C	38.7 – 39.3°C	39.4 – 40.5°C	> 40.5°C
Pain (indicate body site)<br>DO NOT use for pain due to injection (See Injection Site Reactions: Injection site pain)	Pain causing no or minimal interference with usual social & functional activities	Pain causing greater than minimal interference with usual social & functional activities	Pain causing inability to perform usual social & functional activities	Disabling pain causing inability to perform basic self-care functions OR Hospitalization (other than emergency room visit) indicated
Unintentional weight loss	NA	5 – 9% loss in body weight from baseline	10 – 19% loss in body weight from baseline	≥ 20% loss in body weight from baseline OR Aggressive intervention indicated [e.g., tube feeding or total parenteral nutrition (TPN)]

CLINICAL				
PARAMETER	GRADE 1	GRADE 2	GRADE 3	GRADE 4
**INFECTION**				
Infection (any other than HIV infection)	Localized, no systemic antimicrobial treatment indicated AND Symptoms causing no or minimal interference with usual social & functional activities	Systemic antimicrobial treatment indicated OR Symptoms causing greater than minimal interference with usual social & functional activities	Systemic antimicrobial treatment indicated AND Symptoms causing inability to perform usual social & functional activities OR Operative intervention (other than simple incision and drainage) indicated	Life-threatening consequences (e.g., septic shock)
**INJECTION SITE REACTIONS**				
Injection site pain (pain without touching)<br>Or<br>Tenderness (pain when area is touched)	Pain/tenderness causing no or minimal limitation of use of limb	Pain/tenderness limiting use of limb OR Pain/tenderness causing greater than minimal interference with usual social & functional activities	Pain/tenderness causing inability to perform usual social & functional activities	Pain/tenderness causing inability to perform basic self-care function OR Hospitalization (other than emergency room visit) indicated for management of pain/tenderness
Injection site reaction (localized)				
**Adult**	Erythema OR Induration of 5x5 cm – 9x9 cm (or 25 cm^2 – 81cm^2)	Erythema OR Induration OR Edema > 9 cm any diameter (or > 81 cm^2)	Ulceration OR Secondary infection OR Phlebitis OR Sterile abscess OR Drainage	Necrosis (involving dermis and deeper tissue)
Pruritis associated with injection<br>See also Skin: Pruritis (itching - no skin lesions)	Itching localized to injection site AND Relieved spontaneously or with < 48 hours treatment	Itching beyond the injection site but not generalized OR Itching localized to injection site requiring ≥ 48 hours treatment	Generalized itching causing inability to perform usual social & functional activities	NA
**SKIN – DERMATOLOGICAL**				
Alopecia	Thinning detectable by study participant (or by caregiver for young children and disabled adults)	Thinning or patchy hair loss detectable by health care provider	Complete hair loss	NA
Cutaneous reaction – rash	Localized macular rash	Diffuse macular, maculopapular, or morbilliform rash OR Target lesions	Diffuse macular, maculopapular, or morbilliform rash with vesicles or limited number of bullae OR Superficial ulcerations of mucous membrane limited to one site	Extensive or generalized bullous lesions OR Stevens-Johnson syndrome OR Ulceration of mucous membrane involving two or more distinct mucosal sites OR Toxic epidermal necrolysis (TEN)
Hyperpigmentation	Slight or localized	Marked or generalized	NA	NA
Hypopigmentation	Slight or localized	Marked or generalized	NA	NA

CLINICAL				
**PARAMETER**	**GRADE 1**	**GRADE 2**	**GRADE 3**	**GRADE 4**
Pruritis (itching – no skin lesions)<br>(See also Injection Site Reactions: Pruritis associated with injection)	Itching causing no or minimal interference with usual social & functional activities	Itching causing greater than minimal interference with usual social & functional activities	Itching causing inability to perform usual social & functional activities	NA
**CARDIOVASCULAR**				
Cardiac arrhythmia (general)<br>(By ECG or physical exam)	Asymptomatic AND No intervention indicated	Asymptomatic AND Non-urgent medical intervention indicated	Symptomatic, non-life-threatening AND Non-urgent medical intervention indicated	Life-threatening arrhythmia OR Urgent intervention indicated
Cardiac-ischemia/infarction	NA	NA	Symptomatic ischemia (stable angina) OR Testing consistent with ischemia	Unstable angina OR Acute myocardial infarction
Hemorrhage (significant acute blood loss)	NA	Symptomatic AND No transfusion indicated	Symptomatic AND Transfusion of ≤ 2 units packed RBCs (for children ≤ 10 cc/kg) indicated	Life-threatening hypotension OR Transfusion of > 2 units packed RBCs (for children > 10 cc/kg) indicated
Hypertension				
**Adult**<br>(with repeat testing at same visit)	> 140 – 159 mmHg systolic<br>OR<br>> 90 – 99 mmHg diastolic	> 160 – 179 mmHg systolic<br>OR<br>> 100 – 109 mmHg diastolic	> 180 mmHg systolic<br>OR<br>> 110 mmHg diastolic	Life-threatening consequences (e.g., malignant hypertension) OR Hospitalization indicated (other than emergency room visit)
Hypotension	NA	Symptomatic, corrected with oral fluid replacement	Symptomatic, IV fluids indicated	Shock requiring use of vasopressors or mechanical assistance to maintain blood pressure
Pericardial effusion	Asymptomatic, small effusion requiring no intervention	Asymptomatic, moderate or larger effusion requiring no intervention	Effusion with non-life threatening physiologic consequences OR Effusion with non-urgent intervention indicated	Life-threatening consequences (e.g., tamponade) OR Urgent intervention indicated
Prolonged PR interval				
**Adult**	PR interval 0.21 – 0.25 sec	PR interval > 0.25 sec	Type II 2nd degree AV block OR Ventricular pause > 3.0 sec	Complete AV block

CLINICAL				
**PARAMETER**	**GRADE 1**	**GRADE 2**	**GRADE 3**	**GRADE 4**
Prolonged QTc				
**Adult**	Asymptomatic, QTc interval 0.45 – 0.47 sec OR Increase interval < 0.03 sec above baseline	Asymptomatic, QTc interval 0.48 – 0.49 sec OR Increase in interval 0.03 – 0.05 sec above baseline	Asymptomatic, QTc interval ≥ 0.50 sec OR Increase in interval ≥ 0.06 sec above baseline	Life-threatening consequences, e.g. Torsade de pointes or other associated serious ventricular dysrhythmia
Thrombosis/embolism	NA	Deep vein thrombosis AND No intervention indicated (e.g., anticoagulation, lysis filter, invasive procedure)	Deep vein thrombosis AND Intervention indicated (e.g., anticoagulation, lysis filter, invasive procedure)	Embolic event (e.g., pulmonary embolism, life-threatening thrombus)
Vasovagal episode (associated with a procedure of any kind)	Present without loss of consciousness	Present with transient loss of consciousness	NA	NA
Ventricular dysfunction (congestive heart failure)	NA	Asymptomatic diagnostic finding AND intervention indicated	New onset with symptoms OR Worsening symptomatic congestive heart failure	Life-threatening congestive heart failure
**GASTROINTESTINAL**				
Anorexia	Loss of appetite without decreased oral intake	Loss of appetite associated with decreased oral intake without significant weight loss	Loss of appetite associated with significant weight loss	Life-threatening consequences OR Aggressive intervention indicated [e.g., tube feeding or total parenteral nutrition (TPN)]
Ascites	Asymptomatic	Symptomatic AND Intervention indicated (e.g., diuretics or therapeutic paracentesis)	Symptomatic despite intervention	Life-threatening consequences
Cholecystitis	NA	Symptomatic AND Medical intervention indicated	Radiologic, endoscopic, or operative intervention indicated	Life-threatening consequences (e.g., sepsis or perforation)
Constipation	NA	Persistent constipation requiring regular use of dietary modifications, laxatives, or enemas	Obstipation with manual evacuation indicated	Life-threatening consequences (e.g., obstruction)
Diarrhea				
**Adult**	Transient or intermittent episodes of unformed stools OR Increase of ≤ 3 stools over baseline per 24-hour period	Persistent episodes of unformed to watery stools OR Increase of 4 – 6 stools over baseline per 24-hour period	Bloody diarrhea OR Increase of ≥ 7 stools per 24-hour period OR IV fluid replacement indicated	Life-threatening consequences (e.g., hypotensive shock)

CLINICAL				
**PARAMETER**	**GRADE 1**	**GRADE 2**	**GRADE 3**	**GRADE 4**
Dysphagia-Odynophagia	Symptomatic but able to eat usual diet	Symptoms causing altered dietary intake without medical intervention indicated	Symptoms causing severely altered dietary intake with medical intervention indicated	Life-threatening reduction in oral intake
Mucositis/stomatitis (clinical exam)<br>Indicate site (e.g., larynx, oral)	Erythema of the mucosa	Patchy pseudomembranes or ulcerations	Confluent pseudomembranes or ulcerations OR Mucosal bleeding with minor trauma	Tissue necrosis OR Diffuse spontaneous mucosal bleeding OR Life-threatening consequences (e.g., aspiration, choking)
Nausea	Transient (< 24 hours) or intermittent nausea with no or minimal interference with oral intake	Persistent nausea resulting in decreased oral intake for 24 – 48 hours	Persistent nausea resulting in minimal oral intake for > 48 hours OR Aggressive rehydration indicated (e.g., IV fluids)	Life-threatening consequences (e.g., hypotensive shock)
Pancreatitis	NA	Symptomatic AND Hospitalization not indicated (other than emergency room visit)	Symptomatic AND Hospitalization indicated (other than emergency room visit)	Life-threatening consequences (e.g., circulatory failure, hemorrhage, sepsis)
Proctitis (functional-symptomatic)	Rectal discomfort AND No intervention indicated	Symptoms causing greater than minimal interference with usual social & functional activities OR Medical intervention indicated	Symptoms causing inability to perform usual social & functional activities OR Operative intervention indicated	Life-threatening consequences (e.g., perforation)
Vomiting	Transient or intermittent vomiting with no or minimal interference with oral intake	Frequent episodes of vomiting with no or mild dehydration	Persistent vomiting resulting in orthostatic hypotension OR Aggressive rehydration indicated (e.g., IV fluids)	Life-threatening consequences (e.g., hypotensive shock)
**NEUROLOGIC**				
Alteration in personality-behavior or in mood (e.g., agitation, anxiety, depression, mania, psychosis)	Alteration causing no or minimal interference with usual social & functional activities	Alteration causing greater than minimal interference with usual social & functional activities	Alteration causing inability to perform usual social & functional activities	Behavior potentially harmful to self or others (e.g., suicidal and homicidal ideation or attempt, acute psychosis) OR Causing inability to perform basic self-care functions
Altered Mental Status<br>For Dementia, see Cognitive and behavioral/attentional disturbance (including dementia and attention deficit disorder)	Changes causing no or minimal interference with usual social & functional activities	Mild lethargy or somnolence causing greater than minimal interference with usual social & functional activities	Confusion, memory impairment, lethargy, or somnolence causing inability to perform usual social & functional activities	Delirium OR obtundation, OR coma

CLINICAL				
**PARAMETER**	**GRADE 1**	**GRADE 2**	**GRADE 3**	**GRADE 4**
Ataxia	Asymptomatic ataxia detectable on exam OR Minimal ataxia causing no or minimal interference with usual social & functional activities	Symptomatic ataxia causing greater than minimal interference with usual social & functional activities	Symptomatic ataxia causing inability to perform usual social & functional activities	Disabling ataxia causing inability to perform basic self-care functions
Cognitive and behavioral/attentional disturbance (including dementia and attention deficit disorder)	Disability causing no or minimal interference with usual social & functional activities OR Specialized resources not indicated	Disability causing greater than minimal interference with usual social & functional activities OR Specialized resources on part-time basis indicated	Disability causing inability to perform usual social & functional activities OR Specialized resources on a full-time basis indicated	Disability causing inability to perform basic self-care functions OR Institutionalization indicated
CNS ischemia (acute)	NA	NA	Transient ischemic attack	Cerebral vascular accident (CVA, stroke) with neurological deficit
Headache	Symptoms causing no or minimal interference with usual social & functional activities	Symptoms causing greater than minimal interference with usual social & functional activities	Symptoms causing inability to perform usual social & functional activities	Symptoms causing inability to perform basic self-care functions OR Hospitalization indicated (other than emergency room visit) OR Headache with significant impairment of alertness or other neurologic function
Insomnia	NA	Difficulty sleeping causing greater than minimal interference with usual social & functional activities	Difficulty sleeping causing inability to perform usual social & functional activities	Disabling insomnia causing inability to perform basic self-care functions
Neuromuscular weakness (including myopathy & neuropathy)	Asymptomatic with decreased strength on exam OR Minimal muscle weakness causing no or minimal interference with usual social & functional activities	Muscle weakness causing greater than minimal interference with usual social & functional activities	Muscle weakness causing inability to perform usual social & functional activities	Disabling muscle weakness causing inability to perform basic self-care functions OR Respiratory muscle weakness impairing ventilation
Neurosensory alteration (including paresthesia and painful neuropathy)	Asymptomatic with sensory alteration on exam or minimal paresthesia causing no or minimal interference with usual social & functional activities	Sensory alteration or paresthesia causing greater than minimal interference with usual social & functional activities	Sensory alteration or paresthesia causing inability to perform usual social & functional activities	Disabling sensory alteration or paresthesia causing inability to perform basic self-care functions
Seizure: (new onset) **Adult**	NA	1 seizure	2 – 4 seizures	Seizures of any kind which are prolonged, repetitive (e.g., status epilepticus), or difficult to control (e.g., refractory epilepsy)

CLINICAL				
**PARAMETER**	**GRADE 1**	**GRADE 2**	**GRADE 3**	**GRADE 4**
Seizure: (known pre-existing seizure disorder) **– Adult** For worsening of existing epilepsy the grades should be based on an increase from previous level of control to any of these levels.	NA	Increased frequency of pre-existing seizures (non-repetitive) without change in seizure character OR Infrequent break-through seizures while on stable medication in a previously controlled seizure disorder	Change in seizure character from baseline either in duration or quality (e.g., severity or focality)	Seizures of any kind which are prolonged, repetitive (e.g., status epilepticus), or difficult to control (e.g., refractory epilepsy)
Syncope (not associated with a procedure)	NA	Present	NA	NA
Vertigo	Vertigo causing no or minimal interference with usual social & functional activities	Vertigo causing greater than minimal interference with usual social & functional activities	Vertigo causing inability to perform usual social & functional activities	Disabling vertigo causing inability to perform basic self-care functions
**RESPIRATORY**				
Bronchospasm (acute)	FEV1 or peak flow reduced to 70 – 80%	FEV1 or peak flow 50 – 69%	FEV1 or peak flow 25 – 49%	Cyanosis OR FEV1 or peak flow < 25% OR Intubation
Dyspnea or respiratory distress				
**Adult**	Dyspnea on exertion with no or minimal interference with usual social & functional activities	Dyspnea on exertion causing greater than minimal interference with usual social & functional activities	Dyspnea at rest causing inability to perform usual social & functional activities	Respiratory failure with ventilatory support indicated
**MUSCULOSKELETAL**				
Arthralgia	Joint pain causing no or minimal interference with usual social & functional activities	Joint pain causing greater than minimal interference with usual social & functional activities	Joint pain causing inability to perform usual social & functional activities	Disabling joint pain causing inability to perform basic self-care functions
Arthritis	Stiffness or joint swelling causing no or minimal interference with usual social & functional activities	Stiffness or joint swelling causing greater than minimal interference with usual social & functional activities	Stiffness or joint swelling causing inability to perform usual social & functional activities	Disabling joint stiffness or swelling causing inability to perform basic self-care functions
Bone Mineral Loss				
**Adult**	BMD t-score -2.5 to -1.0	BMD t-score < -2.5	Pathological fracture (including loss of vertebral height)	Pathologic fracture causing life-threatening consequences
Myalgia (non-injection site)	Muscle pain causing no or minimal interference with usual social & functional activities	Muscle pain causing greater than minimal interference with usual social & functional activities	Muscle pain causing inability to perform usual social & functional activities	Disabling muscle pain causing inability to perform basic self-care functions

CLINICAL				
**PARAMETER**	**GRADE 1**	**GRADE 2**	**GRADE 3**	**GRADE 4**
Osteonecrosis	NA	Asymptomatic with radiographic findings AND No operative intervention indicated	Symptomatic bone pain with radiographic findings OR Operative intervention indicated	Disabling bone pain with radiographic findings causing inability to perform basic self-care functions
**GENITOURINARY**				
Inter-menstrual bleeding (IMB)	Spotting observed by participant OR Minimal blood observed during clinical or colposcopic examination	Inter-menstrual bleeding not greater in duration or amount than usual menstrual cycle	Inter-menstrual bleeding greater in duration or amount than usual menstrual cycle	Hemorrhage with life-threatening hypotension OR Operative intervention indicated
Urinary tract obstruction (e.g., stone)	NA	Signs or symptoms of urinary tract obstruction without hydronephrosis or renal dysfunction	Signs or symptoms of urinary tract obstruction with hydronephrosis or renal dysfunction	Obstruction causing life-threatening consequences
**OCULAR/VISUAL**				
Uveitis	Asymptomatic but detectable on exam	Symptomatic anterior uveitis OR Medical intervention indicated	Posterior or pan-uveitis OR Operative intervention indicated	Disabling visual loss in affected eye(s)
Visual changes (from baseline)	Visual changes causing no or minimal interference with usual social & functional activities	Visual changes causing greater than minimal interference with usual social & functional activities	Visual changes causing inability to perform usual social & functional activities	Disabling visual loss in affected eye(s)
**ENDOCRINE/METABOLIC**				
Abnormal fat accumulation (e.g., back of neck, breasts, abdomen)	Detectable by study participant (or by caregiver for young children and disabled adults)	Detectable on physical exam by health care provider	Disfiguring OR Obvious changes on casual visual inspection	NA
Diabetes mellitus	NA	New onset without need to initiate medication OR Modification of current medications to regain glucose control	New onset with initiation of medication indicated OR Diabetes uncontrolled despite treatment modification	Life-threatening consequences (e.g., ketoacidosis, hyperosmolar non-ketotic coma)
Gynecomastia	Detectable by study participant or caregiver (for young children and disabled adults)	Detectable on physical exam by health care provider	Disfiguring OR Obvious on casual visual inspection	NA
Hyperthyroidism	Asymptomatic	Symptomatic causing greater than minimal interference with usual social & functional activities OR Thyroid suppression therapy indicated	Symptoms causing inability to perform usual social & functional activities OR Uncontrolled despite treatment modification	Life-threatening consequences (e.g., thyroid storm)

CLINICAL				
**PARAMETER**	**GRADE 1**	**GRADE 2**	**GRADE 3**	**GRADE 4**
Hypothyroidism	Asymptomatic	Symptomatic causing greater than minimal interference with usual social & functional activities OR Thyroid replacement therapy indicated	Symptoms causing inability to perform usual social & functional activities OR Uncontrolled despite treatment modification	Life-threatening consequences (e.g., myxedema coma)
Lipoatrophy (e.g., fat loss from the face, extremities, buttocks)	Detectable by study participant (or by caregiver for young children and disabled adults)	Detectable on physical exam by health care provider	Disfiguring OR Obvious on casual visual inspection	NA

LABORATORY				
**PARAMETER**	**GRADE 1**	**GRADE 2**	**GRADE 3**	**GRADE 4**
**HEMATOLOGY**	**Standard International Units are listed in italics**			
Absolute CD4+ count **– Adult**	300 – 400/mm^3<br>300 – 400/µL	200 – 299/mm^3<br>200 – 299/µL	100 – 199/mm^3<br>100 – 199/µL	< 100/mm^3<br>< 100/µL
Absolute lymphocyte count **– Adult**	600 – 650/mm^3<br>0.600 x 10^9 – 0.650 x 10^9/L	500 – 599/mm^3<br>0.500 x 10^9 – 0.599 x 10^9/L	350 – 499/mm^3<br>0.350 x 10^9 – 0.499 x 10^9/L	< 350/mm^3<br>< 0.350 x 10^9/L
Absolute neutrophil count (ANC)				
**Adult**	1,000 – 1,300/mm^3<br>1.000 x 10^9 – 1.300 x 10^9/L	750 – 999/mm^3<br>0.750 x 10^9 – 0.999 x 10^9/L	500 – 749/mm^3<br>0.500 x 10^9 – 0.749 x 10^9/L	< 500/mm^3<br>< 0.500 x 10^9/L
Fibrinogen, decreased	100 – 200 mg/dL<br>1.00 – 2.00 g/L<br>OR<br>0.75 – 0.99 x LLN	75 – 99 mg/dL<br>0.75 – 0.99 g/L<br>OR<br>0.50 – 0.74 x LLN	50 – 74 mg/dL<br>0.50 – 0.74 g/L<br>OR<br>0.25 – 0.49 x LLN	< 50 mg/dL<br>< 0.50 g/L<br>OR<br>< 0.25 x LLN<br>OR<br>Associated with gross bleeding
Hemoglobin (Hgb)				
**Adult**	10.0 – 10.9 g/dL<br>1.55 – 1.69 mmol/L<br>OR<br>Any decrease<br>2.5 – 3.4 g/dL<br>0.39 – 0.53 mmol/L	9.0 – 9.9 g/dL<br>1.40 – 1.54 mmol/L<br>OR<br>Any decrease<br>3.5 – 4.4 g/dL<br>0.54 – 0.68 mmol/L	7.0 – 8.9 g/dL<br>1.09 – 1.39 mmol/L<br>OR<br>Any decrease<br>≥ 4.5 g/dL<br>≥ 0.69 mmol/L	< 7.0 g/dL<br>< 1.09 mmol/L
International Normalized Ratio of prothrombin time (INR)	1.1 – 1.5 x ULN	1.6 – 2.0 x ULN	2.1 – 3.0 x ULN	> 3.0 x ULN
Methemoglobin	5.0 – 10.0%	10.1 – 15.0%	15.1 – 20.0%	> 20.0%
Prothrombin Time (PT)	1.1 – 1.25 x ULN	1.26 – 1.50 x ULN	1.51 – 3.00 x ULN	> 3.00 x ULN

CLINICAL				
**PARAMETER**	**GRADE 1**	**GRADE 2**	**GRADE 3**	**GRADE 4**
Partial Thromboplastin Time (PTT)	1.1 – 1.66 x ULN	1.67 – 2.33 x ULN	2.34 – 3.00 x ULN	> 3.00 x ULN
Platelets, decreased	100,000 – 124,999/mm^3<br>100.000 x 10^9 – 124.999 x 10^9/L	50,000 – 99,999/mm^3<br>50.000 x 10^9 – 99.999 x 10^9/L	25,000 – 49,999/mm^3<br>25.000 x 10^9 – 49.999 x 10^9/L	< 25,000/mm^3<br>< 25.000 x 10^9/L
WBC, decreased	2,000 – 2,500/mm^3<br>2.000 x 10^9 – 2.500 x 10^9/L	1,500 – 1,999/mm^3<br>1.500 x 10^9 – 1.999 x 10^9/L	1,000 – 1,499/mm^3<br>1.000 x 10^9 – 1.499 x 10^9/L	< 1,000/mm^3<br>< 1.000 x 10^9/L
**CHEMISTRIES**	**Standard International Units are listed in italics**			
Acidosis	NA	pH < normal, but ≥ 7.3	pH < 7.3 without life-threatening consequences	pH < 7.3 with life-threatening consequences
Albumin, serum, low	3.0 g/dL – < LLN<br>30 g/L – < LLN	2.0 – 2.9 g/dL<br>20 – 29 g/L	< 2.0 g/dL<br>< 20 g/L	NA
Alkaline Phosphatase	1.25 – 2.5 x ULN[†]	2.6 – 5.0 x ULN[†]	5.1 – 10.0 x ULN[†]	> 10.0 x ULN[†]
Alkalosis	NA	pH > normal, but ≤ 7.5	pH > 7.5 without life-threatening consequences	pH > 7.5 with life-threatening consequences
ALT (SGPT)	1.25 – 2.5 x ULN	2.6 – 5.0 x ULN	5.1 – 10.0 x ULN	> 10.0 x ULN
AST (SGOT)	1.25 – 2.5 x ULN	2.6 – 5.0 x ULN	5.1 – 10.0 x ULN	> 10.0 x ULN
Bicarbonate, serum, low	16.0 mEq/L – < LLN<br>16.0 mmol/L – < LLN	11.0 – 15.9 mEq/L<br>11.0 – 15.9 mmol/L	8.0 – 10.9 mEq/L<br>8.0 – 10.9 mmol/L	< 8.0 mEq/L<br>< 8.0 mmol/L
Bilirubin (Total)				
**Adult**	1.1 – 1.5 x ULN	1.6 – 2.5 x ULN	2.6 – 5.0 x ULN	> 5.0 x ULN
Calcium, serum, high (corrected for albumin)				
**Adult**	10.6 – 11.5 mg/dL<br>2.65 – 2.88 mmol/L	11.6 – 12.5 mg/dL<br>2.89 – 3.13 mmol/L	12.6 – 13.5 mg/dL<br>3.14 – 3.38 mmol/L	> 13.5 mg/dL<br>> 3.38 mmol/L
Calcium, serum, low (corrected for albumin)				
**Adult**	7.8 – 8.4 mg/dL<br>1.95 – 2.10 mmol/L	7.0 – 7.7 mg/dL<br>1.75 – 1.94 mmol/L	6.1 – 6.9 mg/dL<br>1.53 – 1.74 mmol/L	< 6.1 mg/dL<br>< 1.53 mmol/L
Cardiac troponin I (cTnI)	NA	NA	NA	Levels consistent with myocardial infarction or unstable angina as defined by the manufacturer
Cardiac troponin T (cTnT)	NA	NA	NA	≥ 0.20 ng/mL<br>OR<br>Levels consistent with myocardial infarction or unstable angina as defined by the manufacturer

CLINICAL				
**PARAMETER**	**GRADE 1**	**GRADE 2**	**GRADE 3**	**GRADE 4**
Cholesterol (fasting)				
**Adult**	200 – 239 mg/dL<br>5.18 – 6.19 mmol/L	240 – 300 mg/dL<br>6.20 – 7.77 mmol/L	> 300 mg/dL<br>> 7.77 mmol/L	NA
Creatine Kinase	3.0 – 5.9 x ULN†	6.0 – 9.9 x ULN†	10.0 – 19.9 x ULN†	≥ 20.0 x ULN†
Creatinine	1.1 – 1.3 x ULN†	1.4 – 1.8 x ULN†	1.9 – 3.4 x ULN†	≥ 3.5 x ULN†
Glucose, serum, high				
Nonfasting	116 – 160 mg/dL<br>6.44 – 8.88 mmol/L	161 – 250 mg/dL<br>8.89 – 13.88 mmol/L	251 – 500 mg/dL<br>13.89 – 27.75 mmol/L	> 500 mg/dL<br>> 27.75 mmol/L
Fasting	110 – 125 mg/dL<br>6.11 – 6.94 mmol/L	126 – 250 mg/dL<br>6.95 – 13.88 mmol/L	251 – 500 mg/dL<br>13.89 – 27.75 mmol/L	> 500 mg/dL<br>> 27.75 mmol/L
Glucose, serum, low				
**Adult**	55 – 64 mg/dL<br>3.05 – 3.55 mmol/L	40 – 54 mg/dL<br>2.22 – 3.06 mmol/L	30 – 39 mg/dL<br>1.67 – 2.23 mmol/L	< 30 mg/dL<br>< 1.67 mmol/L
Lactate	< 2.0 x ULN without acidosis	≥ 2.0 x ULN without acidosis	Increased lactate with pH < 7.3 without life-threatening consequences	Increased lactate with pH < 7.3 with life-threatening consequences
LDL cholesterol (fasting)				
**Adult**	130 – 159 mg/dL<br>3.37 – 4.12 mmol/L	160 – 190 mg/dL<br>4.13 – 4.90 mmol/L	≥ 190 mg/dL<br>≥ 4.91 mmol/L	NA
Lipase	1.1 – 1.5 x ULN	1.6 – 3.0 x ULN	3.1 – 5.0 x ULN	> 5.0 x ULN
Magnesium, serum, low	1.2 – 1.4 mEq/L<br>0.60 – 0.70 mmol/L	0.9 – 1.1 mEq/L<br>0.45 – 0.59 mmol/L	0.6 – 0.8 mEq/L<br>0.30 – 0.44 mmol/L	< 0.60 mEq/L<br>< 0.30 mmol/L
Pancreatic amylase	1.1 – 1.5 x ULN	1.6 – 2.0 x ULN	2.1 – 5.0 x ULN	> 5.0 x ULN
Phosphate, serum, low				
**Adult**	2.5 mg/dL – < LLN<br>0.81 mmol/L – < LLN	2.0 – 2.4 mg/dL<br>0.65 – 0.80 mmol/L	1.0 – 1.9 mg/dL<br>0.32 – 0.64 mmol/L	< 1.00 mg/dL<br>< 0.32 mmol/L
Potassium, serum, high	5.6 – 6.0 mEq/L<br>5.6 – 6.0 mmol/L	6.1 – 6.5 mEq/L<br>6.1 – 6.5 mmol/L	6.6 – 7.0 mEq/L<br>6.6 – 7.0 mmol/L	> 7.0 mEq/L<br>> 7.0 mmol/L
Potassium, serum, low	3.0 – 3.4 mEq/L<br>3.0 – 3.4 mmol/L	2.5 – 2.9 mEq/L<br>2.5 – 2.9 mmol/L	2.0 – 2.4 mEq/L<br>2.0 – 2.4 mmol/L	< 2.0 mEq/L<br>< 2.0 mmol/L
Sodium, serum, high	146 – 150 mEq/L<br>146 – 150 mmol/L	151 – 154 mEq/L<br>151 – 154 mmol/L	155 – 159 mEq/L<br>155 – 159 mmol/L	≥ 160 mEq/L<br>≥ 160 mmol/L
Sodium, serum, low	130 – 135 mEq/L<br>130 – 135 mmol/L	125 – 129 mEq/L<br>125 – 129 mmol/L	121 – 124 mEq/L<br>121 – 124 mmol/L	≤ 120 mEq/L<br>≤ 120 mmol/L
Triglycerides (fasting)	NA	500 – 750 mg/dL<br>5.65 – 8.48 mmol/L	751 – 1,200 mg/dL<br>8.49 – 13.56 mmol/L	> 1,200 mg/dL<br>> 13.56 mmol/L
Uric acid	7.5 – 10.0 mg/dL<br>0.45 – 0.59 mmol/L	10.1 – 12.0 mg/dL<br>0.60 – 0.71 mmol/L	12.1 – 15.0 mg/dL<br>0.72 – 0.89 mmol/L	> 15.0 mg/dL<br>> 0.89 mmol/L
**URINALYSIS**	**Standard International Units are listed in italics**			
Hematuria (microscopic)	6 – 10 RBC/HPF	> 10 RBC/HPF	Gross, with or without clots OR with RBC casts	Transfusion indicated

CLINICAL				
**PARAMETER**	**GRADE 1**	**GRADE 2**	**GRADE 3**	**GRADE 4**
Proteinuria, random collection	1 +	2 – 3 +	4 +	NA
Proteinuria, 24 hour collection				
**Adult**	200 – 999 mg/24 h<br>0.200 – 0.999 g/d	1,000 – 1,999 mg/24 h<br>1.000 – 1.999 g/d	2,000 – 3,500 mg/24 h<br>2.000 – 3.500 g/d	> 3,500 mg/24 h<br>> 3.500 g/d

REFERENCES

[1] Fauce SR, Yang OO, Effros RB. Autologous CD4/CD8 co-culture assay: A physiologically-relevant composite measure of CD8(+) T lymphocyte function in HIV-infected persons. J Immunol Methods, 2007: 327(1-2): 75-81.

[2] UNAIDS. Report on the global AIDS epidemic. 2006

[3] www.naco.nic.in

[4] Mehendale SM, Rodrigues JJ, Brookmeyer RS, Gangakhedkar RR, Divekar AD, Gokhale MR, Risbud AR, Paranjape RS, Shepherd ME, Rompalo AE, et al. Incidence and predictors of human immunodeficiency virus type 1 seroconversion in patients attending sexually transmitted disease clinics in India. J Infect Dis 1995; 172: 1486-91.

[5] Mehendale SM, Shepherd ME, Divekar AD, Gangakhedkar RR, Kamble SS, Menon PA, Yadav R, Risbud AR, Paranjape RS, Gadkari DA, Quinn TC, Bollinger RC, Rodrigues JJ. Evidence for high prevalence & rapid transmission of HIV among individuals attending STD clinics in Pune, India. Indian J Med Res 1996; 104: 327-35.

[6] Gangakhedkar RR, Bentley ME, Divekar AD, Gadkari D, Mehendale SM, Shepherd ME, Bollinger RC, Quinn TC. Spread of HIV infection in married monogamous women in India. JAMA 1997; 278: 2090-2.

[7] Bollinger RC, Brookmeyer RS, Mehendale SM, Paranjape RS, Shepherd ME, Gadkari DA, Quinn TC. Risk factors and clinical presentation of acute primary HIV infection in India. JAMA 1997: 278: 2085-9.

[8] Hawkes S, Santhya KG. Diverse realities: sexually transmitted infections and HIV in India. Sex Transm Infect 2002; 78 Suppl 1: 131-9.

[9] Gadkari DA, Moore D, Sheppard HW, Kulkarni SS, Mehendale SM, Bollinger RC. Transmission of genetically diverse strains of HIV-1 in Pune, India. Indian J Med Res 1998; 107: 1-9.

[10] Mandal D, Jana S, Panda S, Bhattacharya S, Ghosh TC, Bhattacharya SK, Chakrabarti S. Distribution of HIV-1 subtypes in female sex workers of Calcutta, India. Indian J Med Res 2000; 112: 165-72.

[11] Chakrabarti S, Panda S, Chatterjee A, Sarkar S, Manna B, Singh NB, Naik TN, Detels R, Bhattacharya SK. HIV-1 subtypes in injecting drug users & their non-injecting wives in Manipur, India. Indian J Med Res 2000; 111: 189-94.

[12] Sahni AK, Prasad VV, Seth P. Genomic diversity of human immunodeficiency virus type-1 in India. Int J STD AIDS 2002; 13: 115-8.

[13] Excler JL. AIDS vaccine development: perspectives, challenges & hopes. Indian J Med Res. 2005; 4: 568-81.

[14] Graham BS. Clinical trials of HIV vaccines. Annu Rev Med 2002; 53: 207-21.

[15] Dolin R. HIV vaccines for prevention of infection and disease in humans. Infect Dis Clin North Am 2000; 14: 1001-16.

[16] Goulder PJ, Rowland-Jones SL, McMichael AJ, Walker BD. Anti-HIV cellular immunity: recent advances towards vaccine design. AIDS 1999; 13 (Suppl A): S121-S136.

[17] Letvin NL, Barouch DH, Montefiori DC. Prospects for vaccine protection against HIV-1 infection and AIDS. Annu Rev Immunol 2002; 20: 73-99.

[18] Donnelly JJ, Ulmer JB, Shiver JW, Liu MA. DNA vaccines. Annu Rev Immunol 1997; 15:617-48.

[19] Tighe H, Corr M, Roman M, Raz E. Gene vaccination: plasmid DNA is more than just a blueprint. Immunol Today 1998; 19: 89-97.

[20] Barouch DH, Letvin NL. Viral evolution and challenges in the development of HIV vaccines. Vaccine 2002; 20 Suppl 4: A66-8.

21 Excler JL, Plotkin S. The prime-boost concept applied to HIV preventive vaccines. AIDS. 1997; 11 Suppl A: S127-37.

[22] McShane H. Prime-boost immunization strategies for infectious diseases. Curr Opin Mol Ther. 2002; 1: 23-7.

23 Estcourt MJ, Ramsay AJ, Brooks A, Thomson SA, Medveckzy CJ, Ramshaw IA. Prime-boost immunization generates a high frequency, high-avidity CD8(+) cytotoxic T lymphocyte population. Int Immunol. 2002; 1: 31-7.

[24] Tartaglia J, Excler JL, El Habib R, Limbach K, Meignier B, Plotkin S, Klein M. Canarypox virus-based vaccines: prime-boost strategies to induce cell-mediated and humoral immunity against HIV.AIDS Res Hum Retroviruses. 1998; Suppl 3: S291-8.

25 Shu Y, Winfrey S, Yang ZY, Xu L, Rao SS, Srivastava I, Barnett SW, Nabel GJ, Mascola JR. Vaccine. 2007; 8: 1398-408.

[26] Rerks-Ngarm S, Brown AE, Khamboonruang C, Thongcharoen P, Kunasol P. HIV/AIDS preventive vaccine 'prime-boost' phase III trial: foundations and initial lessons learned from Thailand. AIDS. 2006; 11: 1471-9.

[27] Salmon-Ceron D, Excler JL, Sicard D, Blanche P, Finkielstzjen L, Gluckman JC, *et al.* Safety and immunogenicity of a recombinant HIV type 1 glycoprotein 160 boosted by a V3 synthetic peptide in HIV-negative volunteers. AIDS Res Hum Retroviruses. 1995; 12: 1479-86.

28 Pantophlet R, Burton DR. GP120: target for neutralizing HIV-1 antibodies. Annu Rev Immunol. 2006; 24: 739-69.

29 Koff WC, Johnson PR, Watkins DI, Burton DR, Lifson JD, Hasenkrug KJ, *et al*. HIV vaccine design: insights from live attenuated SIV vaccines. Nat Immunol. 2006; 1: 19-23.

30 Emini EA, Koff WC. AIDS/HIV. Developing an AIDS vaccine: need, uncertainty, hope. Science 2004; 5679: 1913-4.

[31] Douek DC, Kwong PD, Nabel GJ. The rational design of an AIDS vaccine. Cell. 2006; 4: 677-81.

[32] Mattapallil JJ, Douek DC, Buckler-White A, Montefiori D, Letvin NL, Nabel GJ, Roederer M. Vaccination preserves CD4 memory T cells during acute simian immunodeficiency virus challenge. J Exp Med. 2006; 6: 1533-41.

[33] Letvin NL, Mascola JR, Sun Y, Gorgone DA, Buzby AP, Xu L, *et al.* Preserved CD4+ central memory T cells and survival in vaccinated SIV-challenged monkeys. Science. 2006; 5779: 1530-3.

[34] Letvin NL. Progress and obstacles in the development of an AIDS vaccine. Nat Rev Immunol. 2006; 12: 930-9.

[35] Gupta SB, Jacobson LP, Margolick JB, Rinaldo CR, Phair JP, Jamieson BD, Mehrotra DV, Robertson MN, Straus WL. Estimating the Benefit of an HIV-1 Vaccine That Reduces Viral Load Set Point. J Infect Dis. 2007; 4: 546-50.

[36] McMichael AJ, Callan M, Appay V, Hanke T, Ogg G, Rowland-Jones S. The dynamics of the cellular immune response to HIV infection: implications for vaccination. Philos Trans R Soc Lond B Biol Sci 2000; 355:1007-11.

[37] McMichael AJ, Rowland-Jones SL. Cellular immune responses to HIV. Nature 2001; 410: 980-7.

[38] Doria-Rose NA, Ohlen C, Polacino P, Pierce CC, Hensel MT, Kuller L, *et al.* Multigene DNA priming-boosting vaccines protect macaques from acute CD4+-T-cell depletion after simian-human immunodeficiency virus SHIV89.6P mucosal challenge. J Virol. 2003; 21: 11563-77.

[39] Wilson NA, Reed J, Napoe GS, Piaskowski S, Szymanski A, Furlott J, *et al.* Vaccine-induced cellular immune responses reduce plasma viral concentrations after repeated low-dose challenge with pathogenic simian immunodeficiency virus SIVmac239. J Virol. 2006; 12: 5875-85.

40 Estcourt MJ, McMichael AJ, Hanke T. DNA vaccines against human immunodeficiency virus type 1. Immunol Rev. 2004; 199: 144-55.

41 Ulmer JB, Wahren B, Liu MA. DNA vaccines: recent technological and clinical advances. Discov Med. 2006; 33:109-12.

42 Kent SJ, Zhao A, Best SJ, Chandler JD, Boyle DB, Ramshaw IA. Enhanced T-cell immunogenicity and protective efficacy of a human immunodeficiency virus type 1 vaccine regimen consisting of consecutive priming with DNA and boosting with recombinant fowlpox virus. J Virol. 1998; 12: 10180-8.

[43] Smith JM, Amara RR, Wyatt LS, Ellenberger DL, Li B, Herndon JG, *et al.* Studies in macaques on cross-clade T cell responses elicited by a DNA/MVA AIDS vaccine, better conservation of CD8 than CD4 T cell responses. AIDS Res Hum Retroviruses. 2005; 2: 140-4.

[44] Hanke T, McMichael AJ, Dorrell L. Clinical experience with plasmid DNA- and modified vaccinia virus Ankara-vectored human immunodeficiency virus type 1 clade A vaccine focusing on T-cell induction. J Gen Virol. 2007; 88(Pt 1): 1-12.

[45] Im EJ, Nkolola JP, di Gleria K, McMichael AJ, Hanke T. Induction of long-lasting multi-specific CD8+ T cells by a four-component DNA-MVA/HIVA-RENTA candidate HIV-1 vaccine in rhesus macaques. Eur J Immunol. 2006; 10: 2574-84.

[46] Cebere I, Dorrell L, McShane H, Simmons A, McCormack S, Schmidt C, *et al.* Phase I clinical trial safety of DNA- and modified virus Ankara-vectored human immunodeficiency virus type 1 (HIV-1) vaccines administered alone and in a prime-boost regime to healthy HIV-1-uninfected volunteers. Vaccine. 2006; 4: 417-25.

[47] Smith JM, Amara RR, McClure HM, Patel M, Sharma S, Yi H, *et al.* Multiprotein HIV type 1 clade B DNA/MVA vaccine: construction, safety, and immunogenicity in Macaques. AIDS Res Hum Retroviruses. 2004; 6: 654-65

48 Goonetilleke N, Moore S, Dally L, Winstone N, Cebere I, Mahmoud A, *et al.* Induction of multifunctional human immunodeficiency virus type 1 (HIV-1)-specific T cells capable of proliferation in healthy subjects by using a prime-boost regimen of DNA- and modified vaccinia virus Ankara-vectored vaccines expressing HIV-1 Gag coupled to CD8+ T-cell epitopes. J Virol. 2006; 10: 4717-28.

49 Dunachie SJ, Walther M, Epstein JE, Keating S, Berthoud T, Andrews L, *et al.* A DNA prime-modified vaccinia virus Ankara boost vaccine encoding thrombospondin-related adhesion protein but not circumsporozoite protein partially protects healthy malaria-naive adults against Plasmodium falciparum sporozoite challenge. Infect Immun. 2006; 10: 5933-42.

[50] Miao J, Li X, Liu Z, Xue C, Bujard H, Cui L. Immune responses in mice induced by prime-boost schemes of the Plasmodium falciparum apical membrane antigen 1 (PfAMA1)-based DNA, protein and recombinant modified vaccinia Ankara vaccines. Vaccine. 2006; (37-39): 6187-98.

[51] Gilbert SC, Moorthy VS, Andrews L, Pathan AA, McConkey SJ, Vuola JM, *et al.* Synergistic DNA-MVA prime-boost vaccination regimes for malaria and tuberculosis. Vaccine. 2006; 21: 4554-61.

[52] McShane H, Hill A. Prime-boost immunisation strategies for tuberculosis. Microbes Infect. 2005; (5-6): 962-7.

[53] Harrop R, John J, Carroll MW. Recombinant viral vectors: cancer vaccines. Adv Drug Deliv Rev. 2006; 8: 931-47.

[54] Lindsey KR, Gritz L, Sherry R, Abati A, Fetsch PA, Goldfeder LC, *et al.* Evaluation of prime/boost regimens using recombinant poxvirus/tyrosinase vaccines for the treatment of patients with metastatic melanoma. Clin Cancer Res. 2006; 8: 2526-37.

[55] Ellenberger D, Otten RA, Li B, Aidoo M, Rodriguez IV, Sariol CA, *et al.* HIV-1 DNA/MVA vaccination reduces the per exposure probability of infection during repeated mucosal SHIV challenges. Virology. 2006; 1: 216-25.

[56] Graham BS, Koup RA, Roederer M, Bailer RT, Enama ME, Moodie Z, *et al.* Vaccine Research Center 004 Study Team. Phase 1 safety and immunogenicity evaluation of a multiclade HIV-1 DNA candidate vaccine. J Infect Dis. 2006; 12: 1650-60.

[57] Catanzaro AT, Koup RA, Roederer M, Bailer RT, Enama ME, Moodie Z, *et al.* Phase 1 safety and immunogenicity evaluation of a multiclade HIV-1 candidate vaccine delivered by a replication-defective recombinant adenovirus vector. J Infect Dis. 2006; 12: 1638-49.

[58] Matano T, Kobayashi M, Igarashi H, Takeda A, Nakamura H, Kano M, et al. Cytotoxic T lymphocyte-based control of simian immunodeficiency virus replication in a preclinical AIDS vaccine trial. J Exp Med. 2004; 12: 1709-18.

[59] Casimiro DR, Bett AJ, Fu TM, Davies ME, Tang A, Wilson KA, *et al.* Heterologous human immunodeficiency virus type 1 priming-boosting immunization strategies involving replication-defective adenovirus and poxvirus vaccine vectors. J Virol. 2004; 20: 11434-8.

[60] Ami Y, Izumi Y, Matsuo K, Someya K, Kanekiyo M, Horibata S, *et al.* Priming-boosting vaccination with recombinant Mycobacterium bovis bacillus Calmette-Guerin and a non-replicating vaccinia virus recombinant leads to long-lasting and effective immunity. J Virol. 2005; 20: 12871-9.

[61] Kelleher AD, Puls RL, Bebbington M, Boyle D, Ffrench R, Kent SJ, *et al.* A randomized, placebo-controlled phase I trial of DNA prime, recombinant fowlpox virus boost prophylactic vaccine for HIV-1. AIDS. 2006; 2: 294-7.

[62] Sandström E, Wahren B, Hejdeman B, Nilsson C, Bråve A, Bratt B, *et al.* Multigene, multiclade HIV-1 plasmid DNA prime and MVA boost is safe and highly immunogenic in healthy human volunteers. AIDS Vaccine 06, Amsterdam, the Netherlands, 29 August – 1 September 2006. [Abstract OA03-02]

[63] Pantaleo G. Late Breaker Oral Presentation - AIDS Vaccine 06, Amsterdam, the Netherlands, 29 August – 1 September 2006. (No abstract available).

[64] DD Ho. Construction, development, and evaluation of matched clade-C/B+ DNA- and MVA-Based HIV-1 vaccine candidates. AIDS Vaccine 06, Amsterdam, the Netherlands, 29 August – 1 September 2006. [Abstract S07-05]

[65] De Rose R, Batten CJ, Smith MZ, Fernandez CS, Peut V, Thomson S, *et al.* Comparative efficacy of subtype AE simian-human immunodeficiency virus priming and boosting vaccines in pigtail macaques. J Virol. 2007; 1: 292-300.

66 Xu J, Ren L, Huang X, Qiu C, Liu Y, Liu Y, Shao Y. Sequential priming and boosting with heterologous HIV immunogens predominantly stimulated T cell immunity against conserved epitopes. AIDS. 2006; 18: 2293-303.

[67] Ramanathan VD, Kumar M, Mahalingam J, Narayanan PR, Solomon S, Boaz M, Stevens G, Sayeed E, Scharton-Kersten T, Seth-Smith M, Verlinde C, Vooijs D, Barin B, Lombardo A, Kochhar S, Excler JL, Fast P. A Phase 1 Study to Evaluate the Safety and Immunogenicity of a Recombinant Modified Vaccinia Ankara Virus (TBC-M4) multigenic HIV subtype C Vaccine. AIDS Vaccine 2007, Seattle WA, USA, August 20-23, 2007, [Abstract P06-02]

[68] DD Ho. Construction, development, and evaluation of matched clade-C/B+ DNA- and MVA-Based HIV-1 vaccine candidates. AIDS Vaccine 06, Amsterdam, the Netherlands, 29 August – 1 September 2006. [Abstract S07-05]

[69] Newman FK, Frey SE, Blevins TP, Mandava M, Bonifacio A, Jr., Yan L, et al. Improved Assay To Detect Neutralizing Antibody following Vaccination with Diluted or Undiluted Vaccinia (Dryvax) Vaccine. J Clin Microbiol 2003 41:3154-7.
